# Photosensitive EGFR-Targeted Nanocarriers for Combined Photodynamic and Local Chemotherapy

**DOI:** 10.3390/pharmaceutics14020405

**Published:** 2022-02-13

**Authors:** Elena de las Heras, M. Lluïsa Sagristá, Montserrat Agut, Santi Nonell

**Affiliations:** 1 Institut Químic de Sarrià, Universitat Ramon Llull, 08017 Barcelona, Spain; elenaherasg@iqs.url.edu (E.d.l.H.); montserrat.agut@iqs.url.edu (M.A.); 2 Departament de Bioquímica i Biomedicina Molecular, Facultat de Biologia, Universitat de Barcelona, 08028 Barcelona, Spain; mlsagrista@ub.edu

**Keywords:** mesoporous silica nanoparticles, photodynamic therapy, chemotherapy, cetuximab, EGFR, singlet oxygen, drug delivery

## Abstract

The major limitation of any cancer therapy lies in the difficulty of precisely controlling the localization of the drug in the tumor cells. To improve this drawback, our study explores the use of actively-targeted chemo-photo-nanocarriers that recognize and bind to epidermal growth factor receptor-overexpressing cells and promote the local on-demand release of the chemotherapeutic agent doxorubicin triggered by light. Our results show that the attachment of high concentrations of doxorubicin to cetuximab-IRDye700DX-mesoporous silica nanoparticles yields efficient and selective photokilling of EGFR-expressing cells mainly through singlet oxygen-induced release of the doxorubicin from the nanocarrier and without any dark toxicity. Therefore, this novel triply functionalized nanosystem is an effective and safe nanodevice for light-triggered on-demand doxorubicin release.

## 1. Introduction

The major drawback of any cancer therapy is the lack of selectivity of the treatment, leading to serious side effects for the patients. This limitation lies in the difficulties of precisely delivering the drug selectively to the tumor tissue.

The light-based cancer therapy photodynamic therapy (PDT) involves the administration of a photosensitizing drug that is not cytotoxic per se, but upon activation with light of a specific wavelength triggers the formation of reactive oxygen species, mostly singlet oxygen (^1^O_2_), which leads to irremediable damage and, consequently, tumor regression [1,2]. The combination of PDT with other therapeutic modalities, such as chemotherapy and immunotherapy, can improve the effectiveness of the treatment and, more importantly, increase its selectivity and, thus, its safety [3,4,5,6,7]. A convenient strategy for delivering different therapies at once is by using nano-sized drug delivery systems.

Among all the smart nanovehicles, mesoporous silica nanoparticles (MSNPs) are ideal nanocarriers for co-delivering different agents to the desired locations due to their various advantageous properties, including (i) the capability of co-delivering high payloads of multiple therapeutic agents; (ii) the easy surface functionalization; and (iii) the desired biological behavior, particularly biocompatibility. For these reasons, MSNPs are expected to be the ideal platform for combined chemo-immuno-phototherapy. Furthermore, to avoid unwanted release of the anti-cancer agent from the nanocarrier and thus improve even more the selectivity of the system, the action of the chemotherapeutic agent can be modulated on-demand by the singlet oxygen generated by PDT [8,9,10,11,12,13]. In this way, PDT would also have the function of controlling drug release in this combinatorial treatment strategy. This control could be achieved by the attachment of the antineoplastic agent to the nanocarrier employing a ^1^O_2_ cleavable linker. 

Previously, our laboratory reported the spatial and temporally controlled delivery of doxorubicin (DOX) from gold nanoclusters grafted with ^1^O_2_-releasable DOX and the photosensitizer protoporphyrin IX (PpIX) [14]. The present work builds upon this strategy. Firstly, the delivery system has been changed to MSNPs for the above-mentioned advantages in combinatorial treatments. Secondly, the PpIX photosensitizer has also been replaced by a phthalocyanine, IRDye700DX, which is endowed with a red-shifted absorption spectrum and higher extinction coefficient for better tissue penetration. Finally, the nanoparticles have been decorated with the active-targeting monoclonal antibody cetuximab (Cet) to selectively kill epidermal growth factor receptor (EGFR)-positive cells.

IRDye700DX is a hydrophilic phthalocyanine that readily forms conjugates with primary and secondary amino groups in targeting moieties, e.g., Cet [15,16,17,18]. An IRDye700DX-Cetconjugate has been given fast-track recognition by regulators in the USA and Japan [19]. Nevertheless, to our knowledge, the delivery of IRDye700DX and Cet by nanoparticles has not been investigated so far. 

Therefore, this work aims to investigate whether IRDye700DX-grafted MSNPs with Cet and DOX improve the photodynamic treatment safety in terms of selectivity thanks to the covalent-grafting of the targeting moiety Cet and the on-demand release of DOX triggered by light.

## 2. Materials and Methods

### 2.1. Materials

3-Mercaptopropionic acid (3-MPA), *cis*-1,2-dichloroethylene, DOX, *N*-Hydroxysuccinimide (NHS), *N*-hydroxysulfosuccinimide (sulfo-NHS), (3-aminopropyl)triethoxysilane (APTES), cetyltrimethylammonium chloride (CTAC), tetraethyl orthosilicate (TEOS), 2′-(4-hydroxyphenyl)-5-(4-methyl-1-piperazinyl)-2,5′-bi-1H-benzimidazole trihydrochloride hydrate (Hoechst 33258), phosphate buffer saline (PBS), *N,N*-diisopropylethylamine (DIPEA), bovine serum albumin (BSA), and 1-(4,5-dimethylthiazol-2-yl)-3,5-diphenylformazan (MTT) were purchased from Sigma-Aldrich Chemical Co. (St. Louis, MO, USA). 1-Ethyl-3-(3-dimethylaminopropyl)carbodiimide hydrochloride (EDC·HCl) was purchased from Tokyo Chemical Industry Co., Ltd. (Tokyo, Japan). Dulbecco’s Modified Eagle’s Medium (DMEM; with 4.5 g/L *d*-glucose and without sodium pyruvate or *l*-glutamine), *l*-glutamine solution (200 mM), penicillin-streptomycin solution, trypsin ethylenediaminetetraacetate (EDTA) solution (solution C (0.05%), EDTA (0.02%) with phenol red), and fetal bovine serum (FBS) were purchased from Biological Industries (Kibbutz Beit Haemek, Israel). Roswell Park Memorial Institute (RPMI) 1640 medium was purchased from Fisher Scientific (Waltham, MA, USA). Cet was purchased from Carbosynth Ltd. (Compton, UK). Succinimidyl carbonate-polyethylene glycol (PEG)-carboxymethyl, MW 5,000 (COOH-PEG_5k_-NHS, **5**) was purchased from Laysan Bio Inc. (Arab, AL, USA). IRDye700DX-NHS was purchased from Li-COR (Lincoln, NE, USA) through Bonsai Lab (Alcobendas, Madrid, Spain). All other chemicals were commercially available reagents of at least analytical grade. Milli-Q water (Millipore Corporation, Bedford, MA, USA), with resistivity of 18 MΩ cm, was used.

### 2.2. Synthesis of Mesoporous Silica Nanoparticles’ Precursors

#### 2.2.1. Synthesis of the ^1^O_2_-Cleavable Linker

3-MPA (4.4 mL, 50.5 mmol, 1.9 eq.) was added dropwise to a solution of NaH (60% dispersion in mineral oil) (4.36 g, 109 mmol) in anhydrous dimethylformamide (DMF; 200 mL) at 0 °C for 10 min and 30 min at room temperature (RT). Then, *cis*-1,2-dichloroethylene (2.0 mL, 26.5 mmol, 1 eq.) was added dropwise. The resulting mixture was stirred for 20 min at RT. Afterward, the reaction was heated up to 70 °C for 18 h. Next, the crude was diluted with water (100 mL) and acidified to pH = 2 with KHSO_4_ 1M. The mixture was extracted with EtOAc (200 mL ×3) and the combined organic layers were washed with brine (200 mL), dried over anhydrous MgSO_4_, and evaporated under reduced pressure. The concentrated residue in diethyl ether (50 mL) was extracted with water (40 mL ×3); the organic layer was washed with brine (40 mL), dried over anhydrous MgSO_4_, and concentrated under reduced pressure. Then, the product was recrystallized with EtOAc/hexane and dried under vacuum to afford the desired diacid (*Z*)-3,3′-(ethene-1,2-diylbis(sulfanediyl)dipropanoic acid (**2**). The compound was isolated with a 30% (1.4279 g, 6.1 mmol) yield. The correct synthesis of the desired product was checked by ^1^H-NMR and ^13^C-NMR.

The physical and spectroscopic data of (*Z*)-3,3′-(ethene-1,2-diylbis(sulfanediyl)dipropanoic acid were identical to those reported in the literature [20] (Figures S1–S4).

#### 2.2.2. Conjugation of Doxorubicin to the ^1^O_2_-Cleavable Linker

A mixture of the cleavable linker (**2**) (0.3 mg, 0.9 μmol, 1 eq), EDC·HCl (0.7 mg, 3.6 μmol, 4 eq) and NHS (0.4 mg, 3.6 μmol, 4 eq) in anhydrous DMF (500 μL) was stirred for 2 h at RT. Then, the mixture was treated dropwise with a solution of DOX (0.5 mg, 0.9 μmol, 1 eq) with DIPEA (1.6 μL) in anhydrous DMF. The resulting reaction mixture was allowed to stir at RT for 24 h. Afterward, the orange product **4** was collected without further purification.

#### 2.2.3. Conjugation between NHS-PEG_5kDa_-COOH and Cet

To a solution of NHS-PEG_5kDa_-COOH in PBS pH = 8.0, Cet was added (1:1 wt NHS-PEG-COOH:Cet). The mixture was stirred for 3 h at RT and was then purified using Amicon ultra 2 mL centrifugal units. To this end, the mixture was first centrifuged (15 min, 7500 g), then washed twice by adding 1 mL of PBS pH = 7.4 and centrifuging again (15 min, 7500 g), and the conjugate **6** was finally collected by flipping the centrifuge unit and centrifuging again (4 min, 1000 g).

Immediately before the preparation of Cet-functionalized nanoparticles, a mixture was prepared with conjugate **6**, EDC·HCl (1:1 wt with PEG), and sulfo-NHS (1:1 wt with PEG) in pH = 6.0 phosphate buffer saline (PBS). The mixture was stirred for 40 min at RT. Afterward, it was purified using Amicon ultra 2 mL centrifugal units. Briefly, the mixture was centrifuged (15 min, 7500 g), then washed twice by adding 1 mL of PBS pH = 6.0 and centrifuging again (15 min, 7500 g), and the conjugate **7** was finally collected by flipping the centrifuge unit and centrifuging again (4 min, 1000 g).

### 2.3. Synthesis and Derivatization of Mesoporous Silica Nanoparticles

#### 2.3.1. IRDye700DX-Loaded Nanoparticles

Synthesis of MSNP1 (amino-functionalized nanoparticles): APTES (15 μL) was added to blank MSNPs (22.5 mg) prepared and dispersed in absolute EtOH (22.5 mL). The flask was kept overnight under stirring at 40 °C. Afterward, the reaction mixture was centrifuged (60 min; 14,000 rpm, 15 °C) and washed three times with EtOH. MSNP1 nanoparticles were stored in DMF at 4 °C.

Synthesis of MSNP2 (IRDye700DX-functionalized nanoparticles): IRDye700DXNHS (0.2 mg) was added to the MSNP1 nanoparticles dispersed in anhydrous DMF (22.5 mL). The mixture was stirred overnight. Then, the reaction mixture was centrifuged and the supernatant discarded. The pellet was washed three times with DMF. MSNP2 nanoparticles were stored in DMF at 4 °C protected from light.

Synthesis of PEGylated MSNP2 nanoparticles: NHS-PEG-COOH (5:2 wt ratio of MSNP2:NHS-PEG-COOH) was added to the MSNP2 nanoparticles dispersed in DMF (20 mL). The mixture was stirred for 24 h in the dark at RT. Then, the reaction mixture was centrifuged (60 min; 14,000 rpm, 15 °C) and washed three times with DMF. IRDye700DX-MSNP nanoparticles were stored in DMF at 4 °C.

Synthesis of IRDye700DX-Cet-MSNP nanoparticles: Conjugate **7** was added to a dispersion of MSNP2 nanoparticles in the same volume of PBS pH = 7.4 (5:2:2 wt MSNPs:PEG:Cetuximab) and stirred overnight at RT. Finally, IRDye700DX-Cet-MSNP nanoparticles were collected without further purification.

#### 2.3.2. Doxorubicin-Loaded Nanoparticles

The DOX conjugate **4**, EDC·HCl (1 eq), and NHS (1 eq) were added to the MSNP2 nanoparticles dispersed in DMF (20 mL). The mixture was allowed to react for 24 h at RT. Afterward, the reaction mixture was centrifuged (60 min; 14,000 rpm, 20 °C) and washed three times with DMF to remove the non-conjugated **4**. MSNP3 nanoparticles were stored in DMF at 4 °C.

MSNP3 nanoparticles were either PEGylated with COOH-PEG_5k_-NHS to obtain the IRDye700DX-DOX-MSNP nanoparticles, or with conjugate **7** to obtain the IRDye700DX-Cet-DOX-MSNP nanoparticles, using the procedures described above. 

### 2.4. Physico-Chemical Characterization of the Nanoparticles

The size, polydispersity index (PDI), and zeta-potential of the nanoparticles was determined by dynamic light scattering using a Zetasizer-Malvern Nano ZS90 (Malvern Instruments Ltd., Worcestershire, UK).

### 2.5. Determination of the Drug Concentration in the Nanoparticles

The concentration of the photosensitizer IRDye700DX and the chemo-drug DOX in the nanoparticle formulations was calculated as the difference between the amount of IRDye700DX or DOX added to the reaction mixtures and the amount recovered from supernatant collected upon centrifugation of the nanoparticles (14,000 rpm, 1 h). The IRDye700DX concentration in the supernatant was determined by comparison with absorbance standard curves obtained under the same conditions. The DOX concentration in the supernatant was determined by comparison with emission standard curves obtained employing the same experimental conditions.

### 2.6. Photo-Responsive Release of Doxorubicin from Nanoparticles

To evaluate the photo-responsive drug release from IRDye700DX-Cet-DOX-MSNP, PBS suspensions of the nanoparticles were irradiated with red light using a Red 670 Device (Red Light Man Ltd., Wigan, Greater Manchester, United Kingdom) (*λ*_em_ = 661 ± 10 nm; irradiance = 70 mW·cm^−2^) at increasing light doses. After irradiation, the released DOX from IRDye700DX-Cet-DOX-MSNP was isolated by centrifugation (20 min; 28,000 rpm). The concentration of DOX from the supernatant was determined by fluorescence spectroscopy (*λ*_exc_ = 475 nm).

### 2.7. Cell Lines and Culture Conditions

In vitro studies were performed using AsPC-1 (human pancreatic adenocarcinoma) and MIA PaCa-2 (human pancreas carcinoma) cell lines. Both are adherent and grow in a monolayer up to confluence after seeding. AsPC-1 cells were cultured in RPMI 1640 medium supplemented with 10% (*v*/*v*) FBS, 1% (*v*/*v*) *l*-glutamine and 1% (*v*/*v*) streptomycin-penicillin. MIA PaCa-2 cells were cultured in DMEM (high glucose) supplemented with 10% (*v*/*v*) FBS, 1% (*v*/*v*) *l*-glutamine, 1% (*v*/*v*) streptomycin-penicillin. Both cell lines were seeded in T-75 flasks and were maintained at 37 °C in an incubator containing 5% CO_2_.

### 2.8. Photophysical Characterization of the Nanoparticles

Absorption spectra were recorded on a double beam Varian Cary 600i UV-Vis-NIR spectrophotometer (Agilent Technologies, Santa Clara, CA, USA). Fluorescence emission spectra were recorded using a Fluoromax-4 spectrofluorometer (Horiba Jobin-Yvon, Edison, NJ, USA). Generation of ^1^O_2_ was studied by time-resolved near-infrared phosphorescence (TRNIR) using a customized PicoQuant Fluotime 200 lifetime system (PicoQuant GmbH, Berlin, Germany). IRDye700DX excitation was achieved using pulsed lasers emitting at either 355 nm or 660 nm. The kinetic parameters governing the production and decay of ^1^O_2_ were determined by fitting Equation (1) to the time-resolved phosphorescence intensity signals at 1275 nm, *S*(*t*):

(1) $$S\left(t\right)=S_{0}\cdot \frac{\tau_{\Delta }}{\tau_{\Delta }-\tau_{T}}\cdot \left(e^{-\frac{t}{\tau_{\Delta }}}-e^{-\frac{t}{\tau_{T}}}\right)$$

where *τ*_T_ and *τ*_∆_ are the lifetimes of the IRDye700DX triplet state and ^1^O_2_, respectively, and *S*_0_ is a quantity proportional to the ^1^O_2_ quantum yield. Data were fitted using the FluoFit 5.0 software (PicoQuant GmbH, Berlin, Germany)

### 2.9. Confocal Microscopy

Qualitative uptake studies in cells were carried out by a Leica TCS SP8 laser-scanning confocal spectral microscope (Leica Microsystems Heidelberg, Mannheim, Germany) with Argon and HeNe lasers attached to a Leica DMi8 S Platform inverted microscope. For visualization of the nanoparticle uptake, images were acquired using an APO 40 objective lens. Images were analyzed using ImageJ software.

### 2.10. Singlet Oxygen Generation in Cell Cultures

A quantity of 100,000 AsPC-1 or MIA PaCa-2 cells was seeded in 12-well plates and incubated at 37 °C (5% CO_2_) for 24 h to achieve 80% confluence. Then, the culture medium was removed and the MSNPs, suspended in complete culture medium, were added. IRDye700DX-Cet-MSNP nanoparticles were employed at 2 μM IRDye700DX concentration. After 24 h of incubation, the particles were removed, and the wells were washed 3 times with PBS. Then, cells were trypsinized. Supplemented culture medium (2 mL) was added, and the cells were centrifuged (1100 rpm, 5 min). The pellet was suspended in 1 mL of complete culture medium without phenol red. Afterward, ^1^O_2_ formation and decay were monitored by time-resolved detection of their phosphorescence at 1275 nm after excitation at 355 nm.

### 2.11. Cellular Uptake Assays

Quantitative: A quantity of 12,000 AsPC-1 or MIA PaCa-2 cells were seeded in black 96-well plates and incubated at 37 °C (5% CO_2_) for 24 h to achieve 80% confluence (for 96-well plates, the working volume is 100 μL). Then, the culture medium was removed and the MSNPs, suspended in complete culture medium, were added. IRDye700DX-MSNP nanoparticles with and without Cet were employed at a concentration of 2 µM IRDye700DX. After the corresponding incubation times at 37 °C, the suspensions were removed and the wells were washed 3 times with PBS. Then, 100 µL of SDS (2%) were added and incubated for 30 additional minutes at 37 °C. Afterward, the plates were shaken and IRDye700DX fluorescence was read in a Synergy H1 Hybrid microplate reader (BioTek Instruments, Inc., Winooski, VT, USA) (*λ*_exc_ = 630 nm; *λ*_em_ = 699 nm). The BCA protein kit (Micro BCATM Protein Assay Kit from Thermo Scientific^TM^) was used for protein correlation after reading the IRDye700DX fluorescence. Thus, 50 µL from each well were transferred to a clear 96-well plate. In addition, 50 µL of the BCA working reagent were added to each well. The plates were shaken for 30 secs and incubated at 37 °C for 1 h. Then, the plates were cooled to RT, and the absorbance of each well was measured at 562 nm with the microplate reader. The amount of protein was calculated by using a standard curve of BSA under the same conditions. Then, the fluorescence of the wells was divided with their correlated BSA concentration. Thus, the cellular uptake of nanoparticles was determined as IRdye700DX fluorescence per µg of protein.

Qualitative: A quantity of 100,000 AsPC-1 cells and 80,000 MIA PaCa-2 cells were seeded in glass cover-slips (keeping them in 12-well plates) and incubated at 37 °C (5% CO_2_) for 24 h to achieve 80% confluence. Then, the culture medium was removed and the MSNPs, suspended in complete culture medium, were added. IRDye700DX-MSNP with and without Cet was employed at 2 µM IRDye700DX concentration. After 24 h of incubation time at 37 °C, the nanoparticles were removed and the wells were washed 3 times with PBS. Then, cells were incubated at 37 °C with complete medium with Hoechst 33258 (1 mg/mL stock was diluted 1:5000 in PBS) for 60 min. Afterward, washes with PBS were performed and cells were fixed adding 400 μL of 4% paraformaldehyde (pH = 7.4) for 30 min. Next, the suspensions were removed and washed twice with PBS. Then, the cover-slips were transferred to glass microscope slides adding a drop of Fluoromount solution (Fluoromount™ Aqueous Mounting Medium) and allowed to dry for 30 min. Intracellular fluorescence of IRDye700DX was observed by confocal microscopy with *λ*_exc_ = 638 nm and *λ*_em_ = 650–700 nm; and false-imaged as red. Hoechst 33259 was excited at 405 nm and false-imaged as blue. For all the samples, the same measuring conditions were used.

### 2.12. In Vitro Dark- and Phototoxicity Assays

A quantity of 12,000 AsPC-1 or 10,000 MIA PaCa-2 cells was seeded in 96-well plates and incubated at 37 °C (5% CO_2_) for 24 h to achieve 80% confluence. Then, the culture medium was removed and the nanosystems suspended in complete culture medium were added to achieve an IRDye700DX concentration of 0.8 µM. Cells without any treatment were used as controls. Cells were incubated in the dark at 37 °C for 24 h. Afterward, the nanoparticles were removed, the wells were washed 3 times with PBS, and supplemented medium was then added. Light-treated plates were irradiated with 30 or 60 J·cm^−2^. Dark controls were kept protected from light for the same irradiation time. For phototoxicity studies, red light from a Red 670 Device (RedLightMan) (*λ*_em_ = 661 ± 10 nm; irradiance = 70 mW·cm^−2^ was used). After irradiation, all the plates were incubated at 37 °C for 24 h. Next, the medium was removed and the cell viability was determined by the MTT assay. To this end, the remaining cells were incubated with 0.1 mg/mL MTT in complete medium for 3 h. Then, the medium was discarded and the purple crystals were solubilized with DMSO. Formazan concentration was determined by absorption at *λ* = 562 nm, recorded in a microplate reader. Cell viability was calculated as the ratio between the absorbance of treated cells and that of non-treated cells.

## 3. Results and Discussion

### 3.1. Synthesis of Precursors

#### 3.1.1. Synthesis of the ^1^O_2_-Cleavable Linker

**Figure 1:**
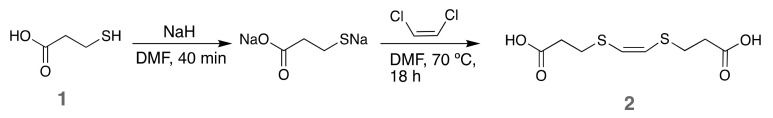
Synthetic procedure for ^1^O_2_-sensitive linker, (*Z*)-3,3′-(ethene-1,2-diylbis(sulfanediyl) dipropanoic acid (**2**).

The synthetic procedure of the ^1^O_2_-sensitive linker (*Z*)-3,3′-(ethene-1,2-diylbis(sulfanediyl) dipropanoic acid (**2**) consisted of two steps (Figure 1): (i) the formation of the disodium salt of 3-MPA (**1**); and (ii) the nucleophilic addition of both thiolate groups to *cis*-1,2-dichloroethylene. Although this procedure was previously described by Wang et al. [20], we introduced some changes. Firstly, when employing NaOMe as a base for the deprotonation of 3-MPA, the overall yield was only 10%. Changing the base to NaH, the overall yield could be tripled. Secondly, we observed that the addition of *cis*-1,2-dichloroethylene in EtOH led to a by-product instead of the corresponding diacid **2**. Thus, we added the *cis*-1,2-dichloroethylene directly onto the disodium salt solution.

#### 3.1.2. Orthogonal Conjugation of Doxorubicin to the ^1^O_2_-Cleavable Linker 

**Figure 2:**
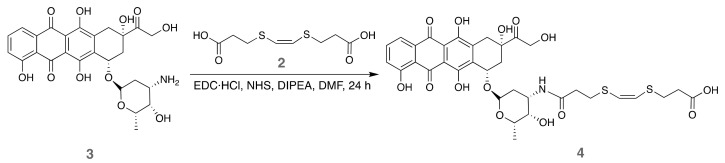
Synthetic procedure for conjugation of DOX to the ^1^O_2_-cleavable linker.

With the required compound **2** in hand, we examined its mono-*N*-acylation with DOX (**3**) by a Steglich reaction (Figure 2). To selectively obtain the orthogonal product, equimolar amounts of DOX and the cleavable linker **2** were used. In the same way, the addition dropwise of DOX over diacid **2** was performed under high-dilution conditions. The orange reaction product obtained was not further purified since the non-reacted DOX and the diamide byproduct would not react with the amino-MSNP, and subsequent washes of the MSNPs removed them.

#### 3.1.3. Conjugation between Cet and PEG

**Figure 3:**
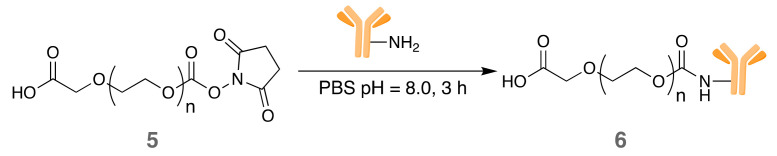
Conjugation between COOH-PEG_5k_-NHS and Cet.

Cet was linked to the PEG chains (**5**) via NHS *N*-acylation reaction in aqueous media (Figure 3). NHS ester-activated compounds react with primary amines in physiologic-to-slightly alkaline conditions (pH 7.2 to 9). The optimum pH range for NHS coupling with proteins is pH 8.0–9.0. At this pH range, amino groups of proteins, i.e., the ε-amino groups of lysines, are unprotonated to a high degree and show high reactivity towards the NHS-ester compound. The pH of the reaction mixture was kept at 8.0 as a compromise between amidation and hydrolysis [21]. The conjugate **6** was purified using an Amicon ultra 2 mL centrifugal unit.

**Figure 4:**
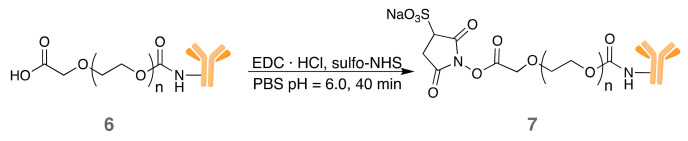
Reaction between *N*-substituted carbodiimide (EDC) and sulfo-NHS with the carboxylic acid group of compound **6** to form the NHS-derivative **7**.

Afterward, a modification of a two-step protocol [22] for the activation of proteins with EDC/sulfo-NHS and subsequent conjugation with amine-containing molecules was performed. The terminal carboxylic acid of PEG of compound **6** was activated with EDC/sulfo-NHS in PBS at pH 6.0 (Figure 4). It is known that carboxylate activation with EDC/NHS occurs most effectively at pH 3.5 to 4.5 [23]. However, the maximal rate of hydrolysis of EDC occurs at acidic pH values, while the stability of the carbodiimides in solution increases at or above pH 6.0. Therefore, to maximize the activation of the carboxylate but increase the stabilization of the active ester intermediate, pH = 6.0 was employed. In addition, at pH 6.0, the amino groups on the protein are protonated and thus less reactive towards the sulfo-NHS esters [21]. Moreover, the active species **7** can be isolated in a reasonable time frame without a significant loss in conjugation potential since the hydrolysis of the sulfo-NHS esters is dramatically slower at slightly acidic pH. Thanks to this fact, the conjugate **7** was purified using an Amicon ultra 2 mL centrifugal unit. Nevertheless, the coupling reaction with amino-functionalized MSNPs was performed on the same day to avoid the hydrolysis problem.

### 3.2. Synthesis and Derivatization of Mesoporous Silica Nanoparticles

#### 3.2.1. IRDye700DX-Cet-MSNP

**Figure 5:**
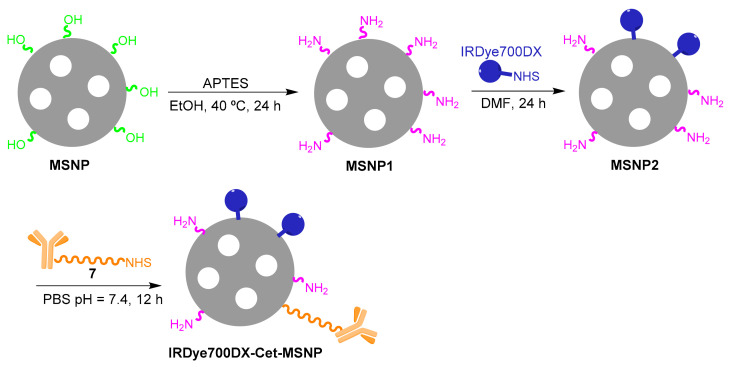
Preparation of IRDye700DX-Cet-MSNP nanoparticles.

The preparation of MSNPs with covalently-bound IRDye700DX and Cet was carried out in three synthetic steps (Figure 5):

1. Modification of the surface of blank MSNPs with amino groups (MSNP1). A high amount of APTES was employed in the reaction to ensure a high degree of surface’s derivatization.
2. Attachment of IRDye700DX-NHS to the MSNP1 nanoparticles via *N*-acylation (MSNP2), whereby the NHS group from IRDye700DX reacted with the MSNP- amino groups.
3. Anchoring of PEG-Cet (conjugate **7**) via *N*-acylation to MSNP2 nanoparticles. The activated carboxyl group of PEG reacted with an amino group on the surface of the MSNP. Unlike in the previous steps, the final product of this reaction was not washed since the resuspension of the pellet required harsh sonication that could have damaged the antibody.

The overall preparation scheme of the non-targeted analogous is shown in Figures S5 and S6. The synthetic route is identical except for the last step, in which COOH-PEG_5k_-NHS **5**, rather than the PEG-Cet conjugate **7**, is attached to the nanoparticles.

#### 3.2.2. IRDye700DX-Cet-DOX-MSNP

**Figure 6:**
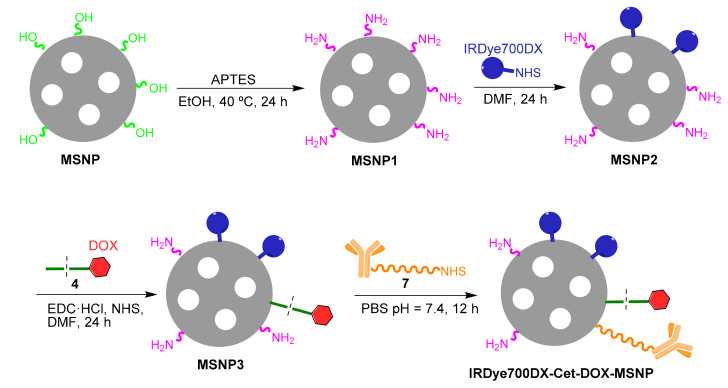
Preparation of IRDye700DX-Cet-DOX-MSNP nanoparticles.

The preparation of MSNPs with covalently-bound IRDye700DX, Cet, and DOX, the latter through the ^1^O_2_-cleavable linker, was similarly carried out in four steps (Figure 6):

1. Modification of the surface of blank MSNPs with amino groups (MSNP1).
2. Attachment of IRDye700DX-NHS to MSNP1 nanoparticles via *N*-acylation (MSNP2).
3. Derivatization of MSNP2 nanoparticles with the DOX conjugate **4** via *N*-acylation (MSNP3). The activated carboxyl of the conjugate **4** reacts with an amino group on the surface of MSNP2.
4. Anchoring of the conjugate PEG-Cet (compound **7**) to the MSNP3 nanoparticles via *N*-acylation.

### 3.3. Physicochemical Characterization of the Nanoparticles

The prepared mesoporous silica nanoparticles were characterized in terms of their physicochemical properties: size, polydispersity index (PDI), zeta-potential, IRDye700DX concentration, DOX concentration, and loading efficiency (Table 1).

After each preparation step, an increase in size and changes in zeta-potential could be observed. These changes are a good indication that the reactions in the nanoparticles had been accomplished. 

The average size of IRDye700DX-Cet-MSNP and IRDye700DX-Cet-DOX-MSNP in aqueous suspension was ~410 nm and ~430 nm, respectively, substantially higher than those of their Cet-free analogs (~243 nm for IRDye700DX-MSNP and ~255 nm for IRDye700DX-DOX-MSNP). Two effects may account for this: On one hand, the attachment of a bulky antibody is expected to increase the nanoparticle diameter, however, no more than a few nanometers. A more likely explanation is nanoparticle aggregation since the Cet-MSNPs were measured in water to avoid denaturalization of the antibody, where they tend to aggregate. On the contrary, Cet-free MSNPs were measured in ethanol, a solvent in which silica nanoparticles are non-aggregated and are thus smaller. 

Consistent with this interpretation, the polydispersity index was smaller than 0.3 for all Cet-free nanoparticles, demonstrating a homogeneous distribution. However, for the Cet-MSNPs, this value was higher, indicating a wide distribution of sizes. 

The zeta-potential of the nanoparticles also changed depending on the moiety introduced. Thus, MSNP1 nanoparticles showed a huge increase in the zeta-potential compared to blank MSNPs due to the attachment of amino groups. Further modification of the MSNP surface induced only minor changes in the zeta potential.

**Figure 7:**
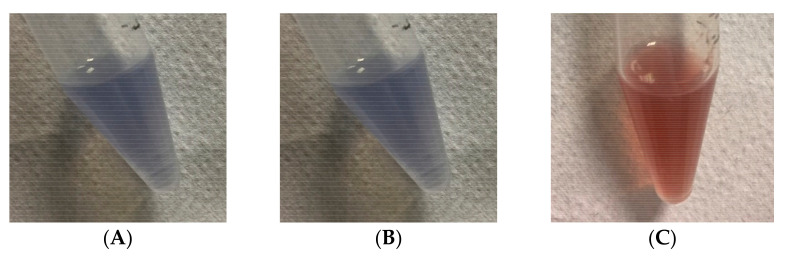
MSNP2 (**A**), MSNP3 18 µM (**B**), and MSNP3 134 µM (**C**) nanoparticle suspensions in dimethyl formamide.

The IRDye700DX and DOX concentrations in the MSNP suspensions were calculated as the difference between the amount added to the reactive mixture and the amount remaining in the supernatants after washing. The results indicate that the attachment of IRDye700DX was 100% efficient. On the other hand, the efficiency of DOX attachment decreased as its concentration in the reactive mixture increased, although the concentration of attached DOX increased (Figure 7). 

### 3.4. Photophysical Characterization of the Nanoparticles

The photophysical behavior of the synthesized nanoparticles was studied by UV-Vis absorption, steady-state fluorescence, and time-resolved near-infrared phosphorescence spectroscopy.

#### 3.4.1. Light Absorption and Steady-State Fluorescence

**Figure 8:**
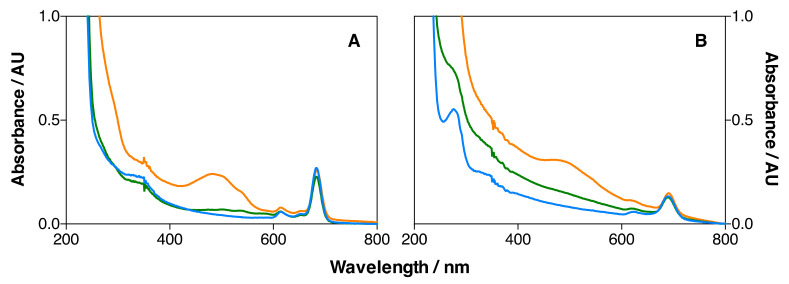
(**A**) Absorption spectra of MSNP2 (blue), MSNP3 (18 µM of DOX) (green) and MSNP3 (134 µM of DOX) (orange) in EtOH; (**B**) absorption spectra of IRDye700DX-Cet-MSNP (blue), IRDye700DX-Cet-DOX-MSNP (18 µM of DOX) (green) and IRDye700DX-Cet-DOX-MSNP (134 µM of DOX) (orange) in dPBS.

The UV-Vis absorption spectra of MSNP2, MSNP3 (18 µM DOX), and MSNP3 (134 µM DOX) nanoparticles dispersed in ethanol are shown in Figure 8A. All show the 693 nm band of IRDye700DX, demonstrating its successful conjugation. In addition, MSNP3 shows a broad band centered at 483 nm, which indicates the presence of DOX in the nanoparticle formulation. 

Likewise, the absorption spectra of IRDye700DX-Cet-MSNP, IRDye700DX-Cet-DOX-MSNP (18 µM DOX) and IRDye700DX-Cet-DOX-MSNP (134 µM DOX) nanoparticles in D_2_O-based PBS (dPBS) are shown in Figure 8B. 

The presence of Cet in these nanoparticle formulations is demonstrated by the band at ~280 nm due to the tryptophan residues of the protein. On the other hand, an increase in scattering can be observed for the Cet nanoparticles, consistent with their larger size. 

**Figure 9:**
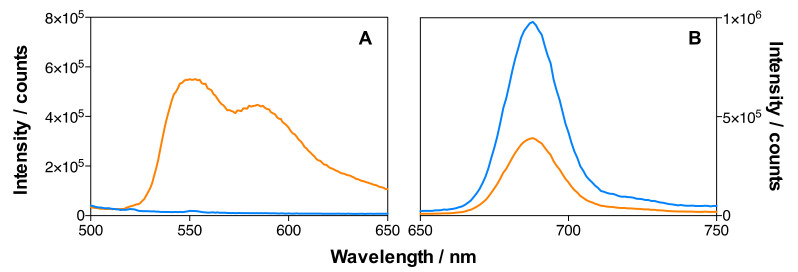
Emission spectra of MSNP2 (blue) and MSNP3 (134 µM of DOX) (orange) in EtOH. The concentration of IRDye700DX was the same in the two MSNP formulations. (**A**) DOX emission, *λ*_exc_ = 475 nm; (**B**) IRDye700DX emission, *λ*_exc_ = 610 nm.

The emission spectra of MSNP2 and MSNP3 (134 µM DOX) nanoparticles in ethanol are shown in Figure 9. The presence of DOX in the MSNP3 nanoparticles is demonstrated by its emission after excitation at 475 nm (Figure 9A), which was absent in the MSNP2 nanoparticles. Despite both formulations having matched concentrations of IRDye700DX, the fluorescence intensity of MSNP3 is only ~40% that of MSNP2 (Figure 9B and Table 2), revealing a quenching effect induced by the binding of DOX. Indeed, separate experiments in aqueous solutions confirm that DOX quenches the fluorescence of IRDye700DX.

**Figure 10:**
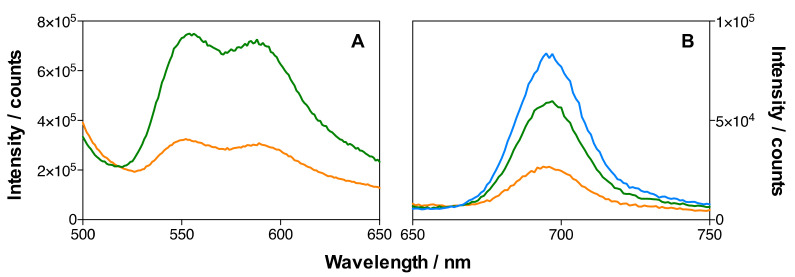
Emission spectra in dPBS of IRDye700DX-Cet-MSNP (blue), IRDye700DX-Cet-DOX-MSNP (18 µM of DOX) (green) and IRDye700DX-Cet-DOX-MSNP (134 µM of DOX) (orange). Same concentration of IRDye700DX in all the MSNP formulations. (**A**) DOX emission, *λ*_exc_ = 475 nm; (**B**) IRDye700DX emission, *λ*_exc_ = 610 nm.

Figure 10 shows the emission spectra of IRDye700DX-Cet-MSNP, IRDye700DX-Cet-DOX-MSNP (18 µM DOX), and IRDye700DX-Cet-DOX-MSNP (134 µM DOX) nanoparticles in dPBS. A decrease in the emission intensity of both DOX (Figure 10A) and IRDye700DX (Figure 10B) can be observed upon increasing the DOX concentration, confirming the quenching observed in ethanol. It is worth noting that quenching is more efficient in dPBS than in ethanol.

#### 3.4.2. Singlet Oxygen Generation

- MSNP2 and MSNP3 nanoparticles

**Figure 11:**
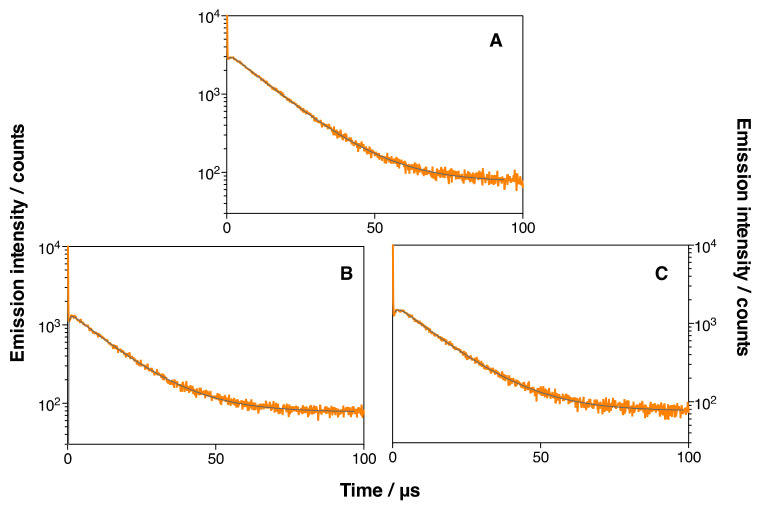
Time-resolved phosphorescence decays of free MSNP2 (**A**), MSNP3 (18 µM DOX) (**B**), and MSNP3 (134 µM DOX) (**C**) in EtOH. *λ*_em_ = 1275 nm. *λ*_exc_ = 660 nm.

Time-resolved near-IR phosphorescence was used to study the generation of ^1^O_2_ by the MSNP2 and MSNP3 nanoparticles in ethanol. The suspensions of the nanoparticles showed the typical rise and decay of ^1^O_2_ upon pulsed laser irradiation (see Equation (1)), with minor differences in lifetimes among all nanosystems tested (Figure 11 and Table 2). The signals grew with a lifetime of ~1 µs, assigned to the formation of ^1^O_2_ and hence to the disappearance of its precursor, the triplet excited state of IRDye700DX. In turn, the signals decayed with a lifetime of ~14 µs, which matches the value of the ^1^O_2_ lifetime in ethanol and was therefore assigned to the decay of this species. 

On the other hand, from the amplitude of the signals, it can be inferred that MSNP3 nanoparticles generate ~50% less ^1^O_2_ than MSNP2, similar to the observed decrease in the IRDye700DX fluorescence emission. This indicates that conjugation of DOX to the nanoparticles by the ^1^O_2_-cleavable linker leads to the quenching of the excited states of IRDye700DX, thereby decreasing the production of ^1^O_2_.

- IRDye700DX-Cet-MSNP and IRDye700DX-Cet-DOX-MSNP nanoparticles

**Figure 12:**
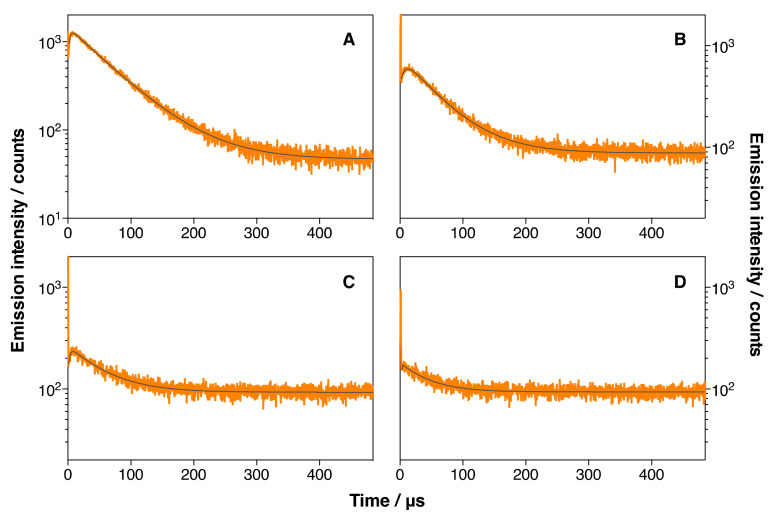
Time-resolved phosphorescence decays of free IRDye700DX (**A**), IRDye700DX-Cet-MSNP (**B**), IRDye700DX-Cet-DOX-MSNP (18 µM DOX) (**C**), and IRDye700DX-Cet-DOX-MSNP (134 µM DOX) (**D**) in dPBS. *λ*_em_ = 1275 nm. *λ*_exc_ = 660 nm.

The generation of singlet oxygen by IRDye700DX-Cet-MSNP and IRDye700DX-Cet-DOX-MSNP nanoparticles was likewise investigated in deuterated PBS (dPBS) suspensions by TRNIR. The results are shown in Figure 12, and the recovered kinetic values are collected in Table 3. Free IRDye700DX gave *τ*_T_ and*τ*_Δ_ values of 2.6 µs and 64 µs, respectively, which are typical values for photosensitizing molecules in aerated dPBS. The results were very different for the nanoparticles. Thus, IRDye700DX-Cet-MSNP nanoparticles showed a longer *τ*_T_ (7.2 µs) and a shorter *τ*_Δ_ (54.7 µs), indicating a shielding effect of the nanoparticle against oxygen quenching of IRDye700DX’s triplet state, and ^1^O_2_ quenching by the nanoparticles. Moreover, a 50% reduction in the ^1^O_2_ generation efficiency relative to the free dye could also be observed. 

Attaching DOX onto the nanoparticles’ surface introduced additional effects: Thus, IRDye700DX-Cet-DOX-MSNP (18 µM DOX) presented a similar *τ*_Δ_ value (53.6 µs) but a shorter *τ*_T_ (2.9 µs). Increasing the concentration of DOX further reduced the two lifetimes (*τ*_Δ_: 42.5 µs and *τ*_T_: 0.3 µs for IRDye700DX-Cet-DOX-MSNP (134 µM DOX)). This indicates that, in addition to quenching the fluorescence of IRDye700DX, i.e., its singlet excited state, conjugation of DOX to the nanoparticles also quenches the triplet excited state of IRDye700DX and ^1^O_2_. 

Consistent with these observations, a decrease in the ^1^O_2_ generation efficiency could also be observed upon attaching DOX to the nanoparticles (Table 3). Remarkably, the decrease was larger than for MSNP2 and MSNP3 and also larger than the decrease in fluorescence yields, supporting the interpretation that attaching DOX to the nanoparticles introduces additional deactivation channels for both the singlet and the triplet excited states of IRDye700DX, leading to lower ^1^O_2_ generation efficiency. 

**Figure 13:**
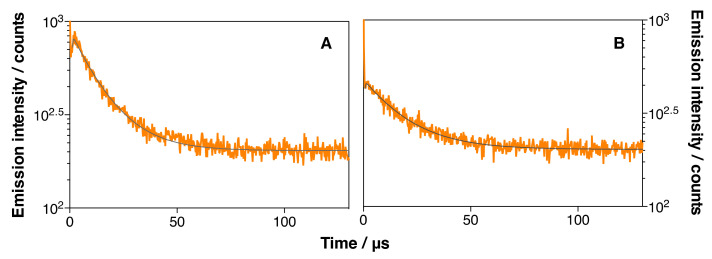
Time-resolved phosphorescence decays of IRDye700DX-Cet-MSNP (**A**) and IRDye700DX-Cet-DOX-MSNP (134 µM DOX) (**B**) in DMF. *λ*_em_ = 1275 nm. *λ*_exc_ = 660 nm.

Further insight was gained by measuring the ^1^O_2_ phosphorescence of IRDye700DX-Cet-MSNP and IRDye700DX-Cet-DOX-MSNP in DMF. The results are shown in Figure 13 and Table 4. No significant differences in the ^1^O_2_ production and decay lifetimes were observed between these two nanoparticles, indicating that the triplet quenching channel is of lesser importance in this less-polar solvent. On the other hand, the quantum yield of ^1^O_2_ production is similar to that observed for the MSNP3 nanoparticles (42% and 51%, respectively, relative to those of the nanoparticles lacking DOX). Taken together, the results indicate that DOX can quench both the singlet and triplet excited states of IRDye700DX in aqueous solvents, while only the singlet excited state is affected in less-polar solvents.

- MSNP4 nanoparticles: Effect of DOX-IRDye700DX distance

A novel MSNP formulation (MSNP4) was synthesized in which DOX was covalently attached to the MSNP through a long PEG_5kDa_ chain. The structure and synthesis of the DOX-PEG conjugate and the MSNP4 nanoparticles are shown in Figures S7 and S8, respectively. The quantum yield of ^1^O_2_ production showed a modest increase of 10% relative to that of the MSNP3 nanoparticles, indicating that the linker had only a minor influence on the quenching of IRDye700DX by DOX.

#### 3.4.3. Photo-Release of DOX 

**Figure 14:**
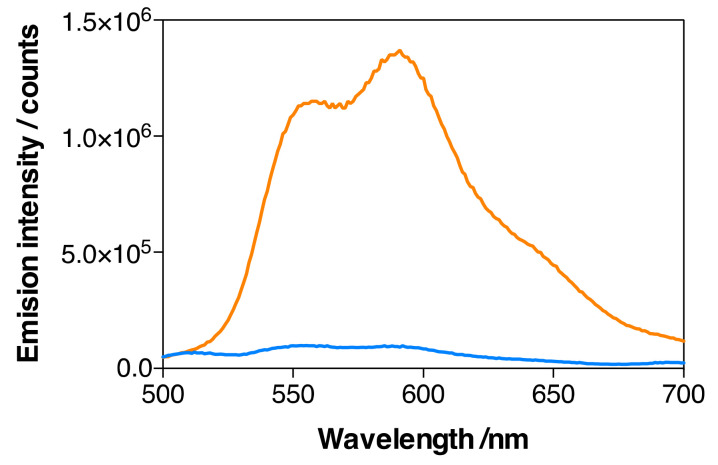
Emission spectra of IRDye700DX-Cet-DOX-MSNP (blue: 18 µM DOX; orange: 134 µM DOX). *λ*_exc_ = 475 nm.

The ability of the nanoparticles to release DOX upon reaction with ^1^O_2_ was next probed. The IRDye700DX-Cet-DOX-MSNP nanoparticles (with either 18 µM or 134 µM DOX) were suspended in PBS and irradiated with a red light dose of 30 J·cm^−2^. After irradiation, the nanoparticles were removed by centrifugation, and the emission spectrum of the supernatants was recorded. Figure 14 shows that DOX was detected in both cases, thereby demonstrating the photo-release of DOX. The efficiency of DOX photo-release was 2% at 18 µM DOX and 12% at 134 µM DOX after a light dose of 30 J·cm^−2^. Concomitant with DOX release, the production of singlet oxygen increased, further confirming the quenching effect of DOX on the excited states of IRDye700DX.

### 3.5. Biological Studies

#### 3.5.1. In Vitro Uptake of IRDye700-Cet-MSNPs

Qualitative uptake: To ascertain the targeting ability of Cetuximab-decorated nanoparticles, AsPC-1, and MIA PaCa-2 cells were incubated with IRDye700DX-MSNP and IRDye700DX-Cet-MSNP at 2 µM of IRDye700DX for 24 h. After incubation, nanoparticles still present in the culture medium were removed by washing. The internalization of the nanoparticles was investigated by studying their fluorescence via confocal microscopy (*λ*_exc_ = 638 nm; *λ*_em_ = 650–700 nm).

**Figure 15:**
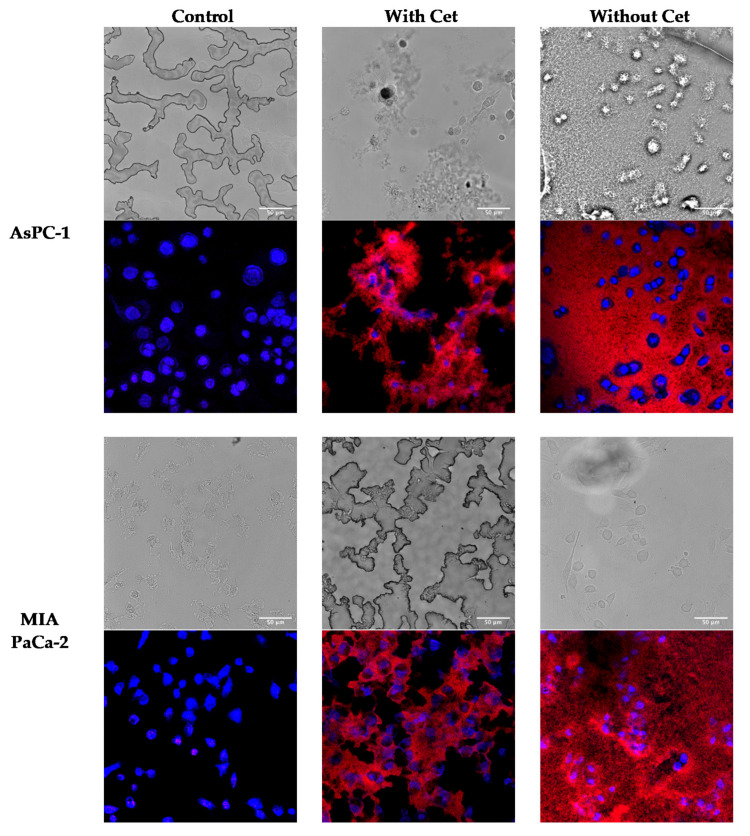
Confocal images of AsPC-1 and MIA PaCa-2 cells after 24 h incubation with IRDye700DX-Cet-MSNP or IRDye700DX-MSNP (2 µM of IRDye700DX) (the same concentration of nanoparticles was incubated). IRDye700DX is shown in red and Hoechst in blue. Wild-field images are shown. Non-treated cells were performed as controls. The scale bar is 50 μm in all pictures.

As shown in Figure 15, AsPC-1 and MIA PaCa-2 cells show a cytosolic distribution of the nanoparticles within the cells. In contrast, non-targeted nanoparticles are not internalized by the cells and appear loosely distributed throughout the coverslip. 

The dramatic difference in uptake between IRDye700DX-MSNP and IRDye700DX-Cet-MSNP is due to both the solubilizing and targeting capacities brought by Cet conjugation. Cet allows the internalization of nanoparticles by receptor-mediated endocytosis; while non-targeted nanoparticles can only enter the cells by less-efficient non-specific pathways. On the other hand, the conjugation of Cet markedly improves their dispersibility. Thus, IRDye700DX-MSNP nanoparticles form aggregates over time that are not uptaken by cells due to their large size. The all-red background observed for non-targeted nanoparticles corresponds to nanoparticle aggregates that precipitated at the bottom of the coverslip and remained attached to the glass despite the washes.

**Figure 16:**
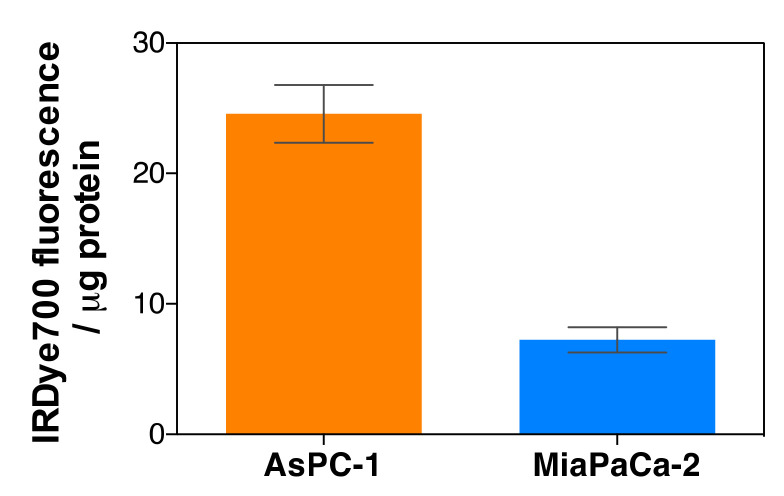
Cellular uptake of IRDye700DX-Cet-MSNP in AsPC-1 and MIA PaCa-2 cells after 24 h of incubation. Cells were treated with the same concentration of IRDye700DX and MSNPs. Values reported are the mean ± SD of at least three independent experiments.

Quantitative uptake: The internalization of MSNPs was investigated in AsPC-1 and MIA PaCa-2 cell lines after 24 h incubation time. Previous studies using fluorescein-labeled nanoparticles had shown that incubation was maximum at 24 h (Figure S9), which is consistent with our previous publication about similar MSNP-Cet nanoparticles [24]. Cells were incubated with IRDye700DX-Cet-MSNP nanoparticles at a concentration of 2 µM of IRDye700DX. After incubation, MSNPs still present in the culture medium were removed by washing. The internalization of the nanoparticles was investigated by studying their fluorescence at *λ*_exc_ = 630 nm and *λ*_em_ = 699 nm. Fluorescence was normalized by the amount of protein present in each sample. The results are summarized in Figure 16. IRDye700DX-Cet-MSNP nanoparticles were internalized more efficiently by AsPC-1 cells than by MIA PaCa-2 cells, which is consistent with the higher level of EGFR expression in the AsPc-1 cell line. 

#### 3.5.2. In Vitro Singlet Oxygen Generation

**Figure 17:**
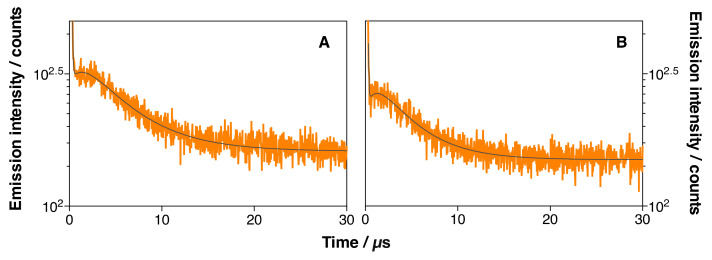
Time-resolved phosphorescence decays for IRDye700DX-Cet-MSNP-treated cells suspensions of AsPC-1 (**A**) and MIA PaCa-2 (**B**) cells. *λ*_exc_ = 1275 nm. *λ*_em_ = 355 nm.

To confirm the generation of ^1^O_2_ in the cells, IRDye700DX-Cet-MSNP-treated cells were studied by time-resolved NIR phosphorescence detection. AsPC-1 and MIA PaCa-2 cells were incubated for 24 h with IRDye700DX-Cet-MSNP and washed twice with PBS to remove non-internalized nanoparticles. Then, cells were trypsinized and resuspended in complete culture medium. Afterward, ^1^O_2_ formation and decay were monitored by time-resolved detection of their phosphorescence at 1275 nm after excitation at 355 nm (Figure 17). Although the same concentration of IRDye700DX-Cet-MSNP had been incubated in both cell lines, the production of ^1^O_2_ was 30% higher in AsPC-1 than in MIA PaCa-2 cells, consistent with the relative uptake of the nanoparticles. 

#### 3.5.3. In Vitro Phototoxicity Studies 

The phototoxic effect of the synthesized nanoparticles, free IRDye700DX, and free DOX was assessed in vitro using the MTT assay.

- Free IRDye700DX and DOX

As shown in Figure S10, free IRDye700DX did not show any cyto- or phototoxicity in AsPC-1 and MIA PaCa-2 cells in the concentration range studied. DOX showed higher cytotoxicity in MIA PaCa-2 cells than in AsPC-1 and no phototoxicity in either cell line.

- IRDye700DX-MSNP and IRDye700DX-Cet-MSNP nanoparticles

**Figure 18:**
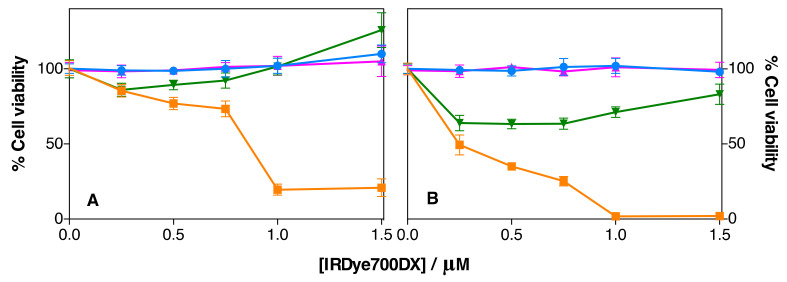
Cytotoxicity studies of IRDye700DX-MSNP nanoparticles (phototoxicity in blue and dark toxicity in pink) and IRDye700DX-Cet-MSNP nanoparticles (phototoxicity in orange and dark toxicity in green) in AsPC-1 (**A**) and MIA PaCa-2 cells (**B**). Values reported are the mean ± SD of at least three independent experiments.

The phototoxicity of IRDye700DX-MSNP and IRDye700DX-Cet-MSNP nanoparticles was investigated in AsPC-1 and MIA PaCa-2 cells. The nanoparticles were diluted in supplemented medium to achieve a range of IRDye700DX concentrations (0.25, 0.5, 0.75, 1, and 1.5 µM) and incubated in the cells for 24 h. Then, cells were irradiated with 30 J·cm^−2^ of red light. As shown in Figure 18, Cet-targeted nanoparticles showed a concentration-dependent phototoxic effect against both cell lines, while Cet-free nanoparticles were not phototoxic, in agreement with their negligible uptake. Therefore, conjugation of the antibody is essential to achieve photodynamic activity by the nanoparticles. 

MIA PaCa-2 cells showed greater susceptibility to photodynamic treatments than AsPC-1 cells, despite their lower uptake. This indicates that the efficacy of targeted-PDT treatments depends not only on the level of EGFR expression of the target cells but also upon their intrinsic biological properties. 

The IRDye700DX-Cet-MSNP nanoparticles showed some degree of dark toxicity, especially in MIA PaCa-2 cells. A control experiment showed that free Cet at the same concentration is not cytotoxic to either cell line (Figure S11).

- IRDye700DX-Cetuximab-DOX-MSNPs nanoparticles

The photodynamic activity of IRDye700DX-Cet-DOX-MSNP nanoparticles was investigated in vitro at four DOX concentration levels (0, 1.5, 2, and 12 µM). An IRDye700DX concentration of 0.8 µM was chosen for all tests since this is the concentration that achieved 50% inhibition in AsPC-1 cells treated by IRDye700DX-Cet-MSNP. The nanoparticles were diluted in supplemented medium and incubated for 24 h with AsPC-1 and MIA PaCa-2 cells. Then, cells were irradiated with 30 J·cm^−2^ of red light. After 24 h or 48 h, the cell proliferation was analyzed by MTT assay. 

**Figure 19:**
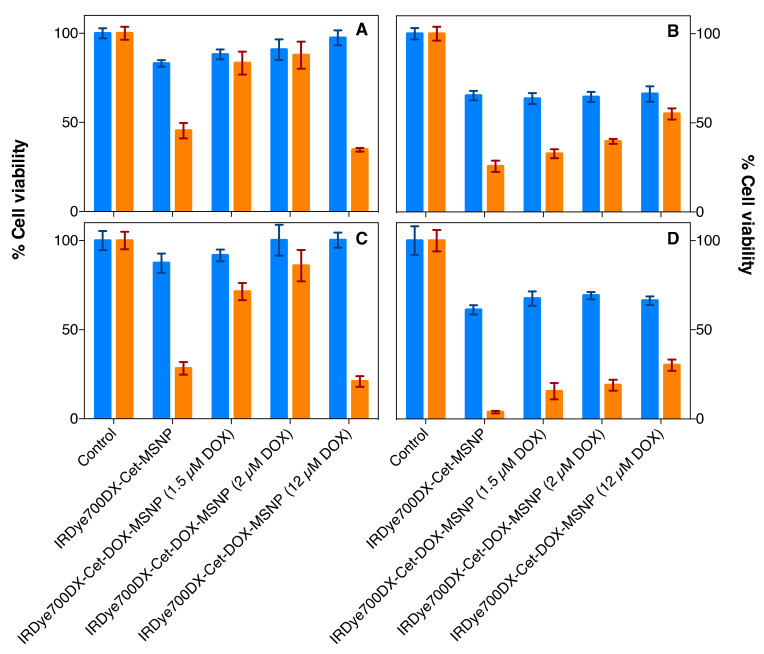
Cytotoxicity studies of IRDye700DX-Cet-MSNP and IRDye700DX-Cet-DOX-MSNP (phototoxicity in orange and dark toxicity in blue) in AsPC-1 (**A**,**C**) and MIA PaCa-2 cells (**B**,**D**); 24 h post-irradiation incubation (**A**,**B**) and 48 h incubation post-irradiation (**C**,**D**). The concentration of IRDye700DX was constant (0.8 µM) for all the nanoparticles’ formulations, as well as the number of MSNPs. Three concentrations of DOX in the nanoparticles were studied, 1.5, 2, and 12 µM. Cells were irradiated with 30 J·cm^−2^. For 48 h post-irradiation incubation, half quantities of cells were seeded than for 24 h. Values reported are the mean ± SD of at least three independent experiments.

The MTT assay results for AsPC-1 cells at 24 h and 48 h are shown in Figure 19A,C, respectively. The first striking observation is that nanoparticles containing 1.5 µM DOX showed lower phototoxicity than DOX-free nanoparticles. This can be rationalized by the reduction of ^1^O_2_ generation efficiency due to quenching of the IRDye700DX excited states by DOX. Consistent with this, the formulation with 2 µM DOX exhibited even lower photodynamic activity. However, the nanoparticles with 12 µM DOX showed a marked decrease in cell viability due to the photorelease of a sufficient amount of DOX in this case. At 48 h, the cell viability is slightly lower, but the results show the same trend.

When the studies were repeated in the MIA PaCa-2, increasing concentrations of DOX led to higher survival, even at 12 µM DOX. This is consistent with the observed lower uptake of the nanoparticles by MIA PaCa-2 cells, which would lead to a concentration of photo-released DOX below the threshold for cytotoxicity. 

Taken together, the results indicate that the photokilling achieved by IRDye700DX-Cet-MSNP nanoparticles is due to the direct action of ^1^O_2_ against cells, while IRDye700DX-Cet-DOX-MSNP nanoparticles kill the cells mainly by the action of photo-released DOX. To confirm this, another batch of AsPC-1 cells was treated with 1.3 µM free DOX and cell viability was assessed after 24 h in the same way. The concentration was arrived at assuming a photorelease of 12% DOX molecules from the uptaken IRDye700DX-Cet-DOX-MSNP nanoparticles, as shown above. The observed decrease of cell viability (75%) was close to that observed for the nanoparticles exposed to light (66%; see Figure 19A).

Wong et al. [8] reported a ^1^O_2_-sensitive nanosystem in which the release of DOX was achieved using a 9,10-dialkoxyanthracene moiety as the cleavable linker. In their study, however, the release of DOX did not increase the phototoxic effect in vitro against HepG2 cells and the dark toxicity of this nanosystem was very large, with a 40% loss of cell viability.

Likewise, Yue et al. [12] reported the formulation of a ROS-triggered theranostic platform based on the Chlorin e6, photosensitizer, camptothecin as the chemotherapeutic drug, and a dithioketal as a ^1^O_2_-cleavable moiety. The introduction of camptothecin to the nanoparticle formulation decreased the cell viability caused a modest 10% additional phototoxicity relative to the formulation without the chemotherapeutic agent.

In a previous publication from our laboratory, we used gold nanoclusters to deliver the photosensitizer protoporphyrin IX and DOX using the same ^1^O_2_-cleavable linker as in this work [14]. The release of DOX caused a larger (60%) cell viability decrease, which is not surprising because the concentration of caged DOX was 2.5-fold higher.

Conjugates between IRDye700DX and Cet are extensively investigated, as is the combination of IRDye700DX and a targeting moiety in nanocarriers [25,26,27]. However, to our knowledge, until today, no research has been published about the combination of these two moieties in nanoparticles. The novel IRDye700DX-Cet-DOX-MSNP nanoparticle formulation is a novel drug delivery system that selectively kills cells with a high level of EGFR expression through the action of light.

## 4. Conclusions

Conjugation of the Cet antibody to PEG-coated MSNPs containing the photosensitizer IRDye700DX improves the aqueous dispersibility of this nanocarrier and enables the selective photokilling of EGFR-expressing cells. DOX can be effectively released from such MSNPs by ^1^O_2_-mediated cleavage of its linker in a light-dose-dependent manner. This novel triply functionalized nanosystem is an effective and safe nanodevice for light-triggered on-demand DOX delivery.

## Figures and Tables

**Table 1:** Characterization of nanoparticles.

Sample	NP Formulation	Size/nm	PDI	Zeta-Potential/mV	[IRDye700DX]/μM	[DOX]/μM	LE
Blank	MSNP	165 ± 3	0.07 ± 0.04 ^a^	−22 ±1	-	-	-
With IRDye700DX and without DOX	MSNP1	165 ± 1	0.03 ± 0.03 ^a^	6 ± 1	-	-	-
	MSNP2	172 ± 2	0.13 ± 0.02 ^a^	6 ± 1	9.2	-	100%
	IRDye700DX-MSNP	243 ± 4	0.11 ± 0.09 ^a^	10 ± 1	9.2	-	-
	IRDye700DX-Cet-MSNP	410 ± 38	0.95 ± 0.10 ^b^	-	9.2	-	-
With IRDye700DX and with releasable DOX	MSNP3	196 ± 3	0.10 ± 0.05 ^a^	8 ± 1	9.2	18.7 21.7 134.4	68% 59% 29%
	IRDye700DX-DOX-MSNP	255 ± 6	0.12 ± 0.04 ^a^	6 ± 1	9.2	18.7 21.7 134.4	-
	IRDye700DX-Cet-DOX-MSNP	430 ± 52	0.89 ± 0.04 ^b^	-	9.2	18.7 21.7 134.4	-

^a^ In ethanol. ^b^ In water. PDI: polydispersity index. LE: loading efficiency.

**Table 2:** Photophysical properties of MSNP2, MSNP3 (18 µM DOX), and MSNP3 (134 µM DOX) suspensions in EtOH. Φ_F_: fluorescence quantum yield; *τ*_T_: IRDye700DX triplet state lifetime; *τ*_Δ_: singlet oxygen lifetime; Φ_Δ_: singlet oxygen quantum yield.

Sample	Φ_F_^a^	*τ*_T_/µs	*τ*_Δ_/µs	Φ_Δ_^a^
MSNP2	1	1.0	14.2	1
MSNP3 (18 µM of DOX)	-	0.9	14.0	0.48
MSNP3 (134 µM of DOX)	0.40	1.2	14.5	0.51

^a^ Relative values.

**Table 3:** Photophysical properties of IRDye700DX, IRDye700DX-Cet-MSNP, IRDye700DX-Cet-DOX-MSNP (18 µM DOX), and IRDye700DX-Cet-DOX-MSNP (134 µM DOX) suspensions in dPBS. Φ_F_: Fluorescence quantum yield; *τ*_T_: Triplet state lifetime; *τ*_Δ_: Singlet oxygen lifetime; Φ_Δ_: Singlet oxygen quantum yield.

Sample	Φ_F_^a^	*τ*_T_/µs	*τ*_Δ_/µs	Φ_Δ_^a^
IRDye700DX	-	2.6	63.8	2.0
IRDye700DX-Cet-MSNP	1	7.2	54.7	1
IRDye700DX-Cet-DOX-MSNP (18 µM of DOX)	0.70	2.9	53.6	0.24
IRDye700DX-Cet-DOX-MSNP (134 µM of DOX)	0.30	0.3	42.5	0.12

^a^ Relative values.

**Table 4:** Singlet oxygen kinetic parameters for IRDye700DX-Cet-MSNP and IRDye700DX-Cet-DOX-MSNP (134 µM DOX) suspensions in DMF.

Sample	*τ*_T_/µs	*τ*_Δ_/µs	Φ_Δ_^a^
IRDye700DX-Cet-MSNP	0.23	17.5	1
IRDye700DX-Cet-DOX-MSNP (134 µM of DOX)	0.29	18.3	0.42

^a^ Relative values.

## Data Availability

The data presented in this study are available on request from the corresponding author.

## Supplementary Materials

The following are available online at https://www.mdpi.com/article/10.3390/pharmaceutics14020405/s1, Figure S1: ^1^H NMR spectra of (*Z*)-3,3′-(ethene-1,2-diylbis(sulfanediyl)dipropanoic acid (**2**), Figure S2: ^13^C NMR spectra of (*Z*)-3,3′-(ethene-1,2-diylbis(sulfanediyl)dipropanoic acid (**2**), Figure S3: gHSQC spectra of (*Z*)-3,3′-(ethene-1,2-diylbis(sulfanediyl)dipropanoic acid (**2**), Figure S4: COSY spectra of (*Z*)-3,3′-(ethene-1,2-diylbis(sulfanediyl)dipropanoic acid (**2**), Figure S5: Preparation of the nanoparticle IRDye700DX-MSNP, Figure S6: Preparation of the nanoparticle IRDye700-DOX-MSNP, Figure S7: Preparation of the DOX-PEG conjugate, Figure S8: Preparation of the MSNP4 nanoparticles with covalently attached IRDye700DX and DOX-PEG_5kDa_, Figure S9: Cellular uptake of FITC-MSNP and FITC-cetuximab-MSNP nanoparticles in AsPC-1 and MIA PaCa-2 cells at different incubation times, Figure S10: Phototoxicity studies of IRDye700DX and doxorubicin in AsPC-1 and MIA PaCa-2 cells, Figure S11: Phototoxicity studies of free Cetuximab in AsPC-1 and MIA PaCa-2 cells, Thermodynamic feasibility of electron-transfer quenching of IRDye700DX by doxorubicin. Refs. [28,29,30] are cited in Supplementary Materials.

## References

[1] Dougherty T.J., Gomer C.J., Henderson B.W., Jori G., Kessel D., Korbelik M., Moan J., Peng Q. (1998). Photodynamic Therapy. JNCI J. Natl. Cancer Inst..

[2] Gunaydin G., Gedik M.E., Ayan S. (2021). Photodynamic Therapy for the Treatment and Diagnosis of Cancer–A Review of the Current Clinical Status. Front. Chem..

[3] Zhang Q., Li L. (2018). Photodynamic combinational therapy in cancer treatment. J. BUON.

[4] Sandland J., Boyle R.W. (2019). Photosensitizer Antibody–Drug Conjugates: Past, Present, and Future. Bioconj. Chem..

[5] Ghosh S., Gul A.R., Xu P., Lee S.Y., Rafique R., Kim Y.H., Park T.J. (2022). Target delivery of photo-triggered nanocarrier for externally activated chemo-photodynamic therapy of prostate cancer. Mater. Today Chem..

[6] Hao Y., Chung C.K., Yu Z., Huis in ‘t Veld R.V., Ossendorp F.A., ten Dijke P., Cruz L.J. (2022). Combinatorial Therapeutic Approaches with Nanomaterial-Based Photodynamic Cancer Therapy. Pharmaceutics.

[7] Liu Z., Xie Z., Li W., Wu X., Jiang X., Li G., Cao L., Zhang D., Wang Q., Xue P. (2021). Photodynamic immunotherapy of cancers based on nanotechnology: Recent advances and future challenges. J. Nanobiotechnol..

[8] Wong R.C.H., Ng D.K.P., Fong W.-P., Lo P.-C. (2020). Glutathione- and light-controlled generation of singlet oxygen for triggering drug release in mesoporous silica nanoparticles. J. Mater. Chem. B.

[9] Lee J., Park J., Singha K., Kim W.J. (2013). Mesoporous silica nanoparticle facilitated drug release through cascade photosensitizer activation and cleavage of singlet oxygen sensitive linker. Chem. Commun..

[10] Yuan Y., Liu J., Liu B. (2014). Conjugated-Polyelectrolyte-Based Polyprodrug: Targeted and Image-Guided Photodynamic and Chemotherapy with On-Demand Drug Release upon Irradiation with a Single Light Source. Angew. Chem. Int. Ed..

[11] Bio M., Rajaputra P., Nkepang G., You Y. (2014). Far-Red Light Activatable, Multifunctional Prodrug for Fluorescence Optical Imaging and Combinational Treatment. J. Med. Chem..

[12] Yue C., Zhang C., Alfranca G., Yang Y., Jiang X., Yang Y., Pan F., de la Fuente J.M., Cui D. (2016). Near-Infrared Light Triggered ROS-activated Theranostic Platform based on Ce6-CPT-UCNPs for Simultaneous Fluorescence Imaging and Chemo-Photodynamic Combined Therapy. Theranostics.

[13] Li J., Cui D., Jiang Y., Huang J., Cheng P., Pu K. (2019). Near-Infrared Photoactivatable Semiconducting Polymer Nanoblockaders for Metastasis-Inhibited Combination Cancer Therapy. Adv. Mater..

[14] Tabero A., Planas O., Gallavardin T., Nieves I., Nonell S., Villanueva A. (2020). Smart Dual-Functionalized Gold Nanoclusters for Spatio-Temporally Controlled Delivery of Combined Chemo- and Photodynamic Therapy. Nanomaterials.

[15] von Felbert V., Bauerschlag D., Maass N., Bräutigam K., Meinhold-Heerlein I., Woitok M., Barth S., Hussain A.F. (2016). A specific photoimmunotheranostics agent to detect and eliminate skin cancer cells expressing EGFR. J. Cancer Res. Clin. Oncol..

[16] Sadraeian M., Bahou C., da Cruz E.F., Janini L.M.R., Sobhie Diaz R., Boyle R.W., Chudasama V., Eduardo Gontijo Guimarães F. (2020). Photoimmunotherapy Using Cationic and Anionic Photosensitizer-Antibody Conjugates against HIV Env-Expressing Cells. Int. J. Mol. Sci..

[17] Boss M., Bos D., Frielink C., Sandker G., Bronkhorst P., van Lith S.A.M., Brom M., Buitinga M., Gotthardt M. (2020). Receptor-Targeted Photodynamic Therapy of Glucagon-Like Peptide 1 Receptor–Positive Lesions. J. Nucl. Med..

[18] Ngen E.J., Chen Y., Azad B.B., Boinapally S., Jacob D., Lisok A., Shen C., Hossain M.S., Jin J., Bhujwalla Z.M. (2021). Prostate-specific membrane antigen (PSMA)-targeted photodynamic therapy enhances the delivery of PSMA-targeted magnetic nanoparticles to PSMA-expressing prostate tumors. Nanotheranostics.

[19] Kobayashi H., Furusawa A., Rosenberg A., Choyke P.L. (2021). Near-infrared photoimmunotherapy of cancer: A new approach that kills cancer cells and enhances anti-cancer host immunity. Int. Immunol..

[20] Wang H., Han R.-L., Yang L.-M., Shi J.-H., Liu Z.-J., Hu Y., Wang Y., Liu S.-J., Gan Y. (2016). Design and Synthesis of Core–Shell–Shell Upconversion Nanoparticles for NIR-Induced Drug Release, Photodynamic Therapy, and Cell Imaging. ACS Appl. Mater. Interfaces.

[21] Hermanson G.T. (2013). Bioconjugate Techniques.

[22] Grabarek Z., Gergely J. (1990). Zero-length crosslinking procedure with the use of active esters. Anal. Biochem..

[23] Nakajima N., Ikada Y. (1995). Mechanism of Amide Formation by Carbodiimide for Bioconjugation in Aqueous Media. Bioconj. Chem..

[24] Er Ö., Colak S.G., Ocakoglu K., Ince M., Bresolí-Obach R., Mora M., Sagristá M.L., Yurt F., Nonell S. (2018). Selective Photokilling of Human Pancreatic Cancer Cells Using Cetuximab-Targeted Mesoporous Silica Nanoparticles for Delivery of Zinc Phthalocyanine. Molecules.

[25] Dou X., Nomoto T., Takemoto H., Matsui M., Tomoda K., Nishiyama N. (2018). Effect of multiple cyclic RGD peptides on tumor accumulation and intratumoral distribution of IRDye 700DX-conjugated polymers. Sci. Rep..

[26] Li F., Zhao Y., Mao C., Kong Y., Ming X. (2017). RGD-Modified Albumin Nanoconjugates for Targeted Delivery of a Porphyrin Photosensitizer. Mol. Pharm..

[27] Deken M.M., Kijanka M.M., Beltrán Hernández I., Slooter M.D., de Bruijn H.S., van Diest P.J., van Bergen En Henegouwen P.M.P., Lowik C.W.G.M., Robinson D.J., Vahrmeijer A.L. (2020). Nanobody-targeted photodynamic therapy induces significant tumor regression of trastuzumab-resistant HER2-positive breast cancer, after a single treatment session. J. Control. Release.

[28] Rehm D., Weller A. (1970). Kinetics of Fluorescence Quenching by Electron and H-Atom Transfer. Isr. J. Chem..

[29] Bandera Y., Burdette M., Shetzline J.A., Jenkins R., Creager S.E., Foulger S. (2016). Synthesis of water soluble axially disubstituted silicon (IV) phthalocyanines with alkyne & azide functionality. Dye Pigment..

[30] Guin P.S., Das S. (2014). Exploration of Electrochemical Intermediates of the Anticancer Drug Doxorubicin Hydrochloride Using Cyclic Voltammetry and Simulation Studies with an Evaluation for Its Interaction with DNA. Int. J. Electrochem..

