# Supplementary material for: Photosensitive EGFR-Targeted Nanocarriers for Combined Photodynamic and Local Chemotherapy
Source: Pharmaceutics. 2022 Feb 13;14(2):405. doi: 10.3390/pharmaceutics14020405 (PMC8880051; doi:10.3390/pharmaceutics14020405)
Supplement: Supplementary file 1 [file pharmaceutics-14-00405-s001.zip › pharmaceutics-1526766-supplementary.pdf]

pharmaceutics MDPI

# Supplementary Materials: Photosensitive EGFR-Targeted Nanocarriers for Combined Photodynamic and Local Chemotherapy

**Elena de las Heras, M. Lluïsa Sagristá, Montserrat Agut and Santi Nonell**

## S1. Spectral data of the singlet oxygen-cleavable linker 2

$^{1}$H NMR, $^{13}$C NMR, gHSQC and COSY spectra of (*Z*)-3, 3′-(ethene-1,2-diylbis(sulfanediyl)dipropanoic acid (2) are shown in Figure S1–S4.

TLC: 0.32 (EtOAc:hexane; 1:1)

$^{1}$H NMR ($CD_3OD$): $\delta$ 6.20 (s, 2H), 2.93 (td, $J$ = 7.0, 0.5 Hz, 4H), 2.61 (td, $J$ = 7.0, 0.5 Hz, 4H).

$^{13}$C NMR ($CD_3OD$): $\delta$ 175.4 (C), 124.9 (CH), 36.2 ($CH_2$), 29.9 ($CH_2$).

IR (KBr) spectroscopy: $\upsilon$, cm$^{-1}$ 3431 (st O-H free), 3300-2500 (st O-H bonded), 3026 (st C-H), 2760 and 2588 (st C-$H_2$), 1639 (st C=O), 1554 (st C=C), 1409 ($\delta$ C-$H_2$), 1198 (st C-O) 642 (*cis* C=C).

MS (EI): *m*/*z* (abundance) $C_8H_{12}O_4S_2$ 236.1 (100).

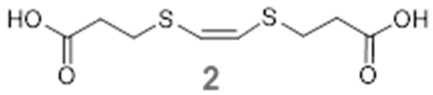


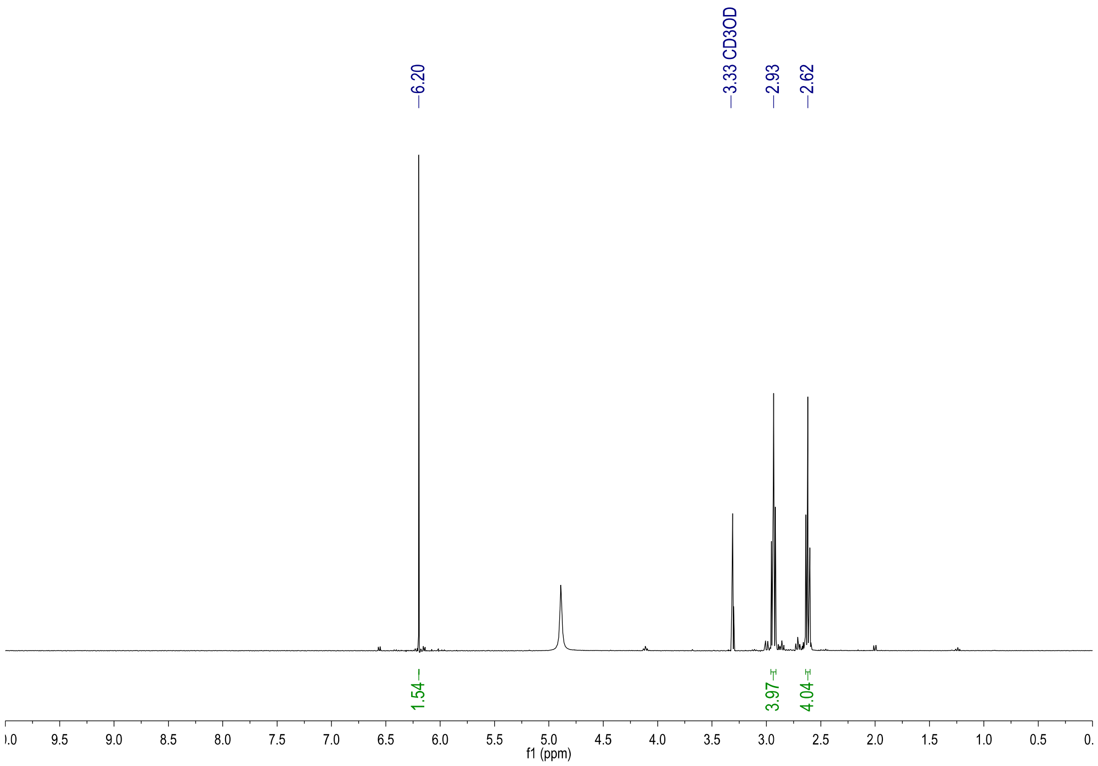


**Figure S1.** $^{1}$H NMR spectra of (*Z*)-3,3′-(ethene-1,2-diylbis(sulfanediyl)dipropanoic acid (**2**).

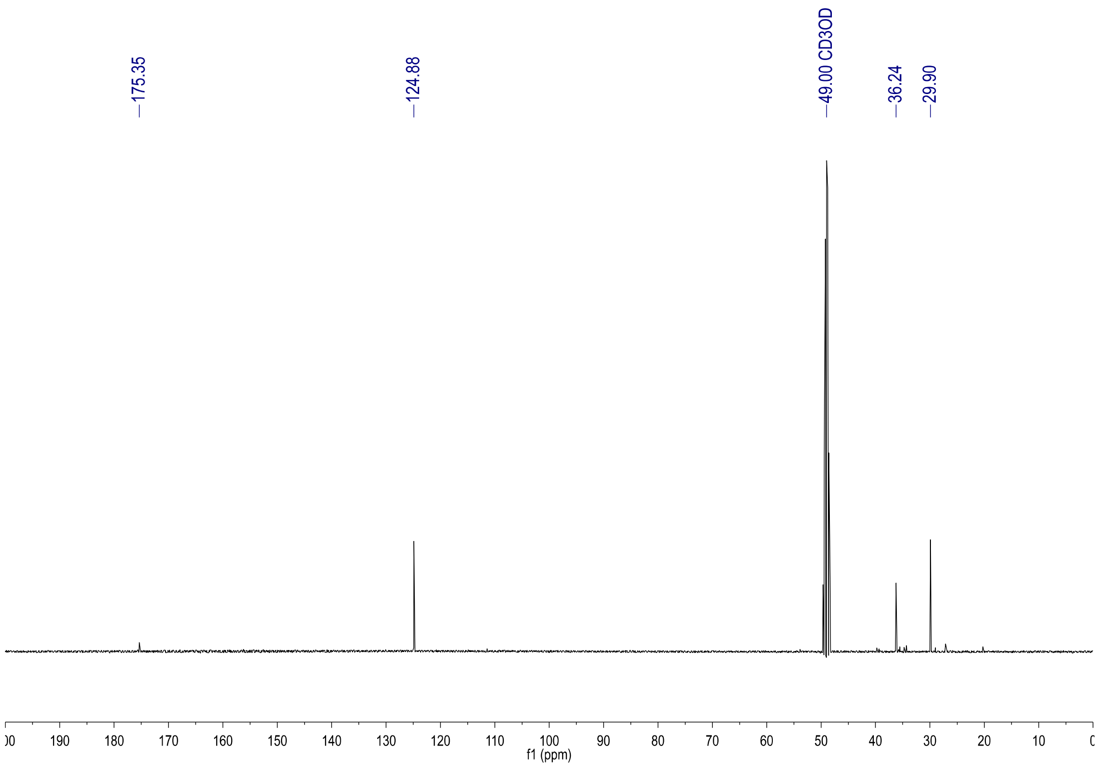


**Figure S2.** $^{13}$C NMR spectra of (*Z*)-3,3′-(ethene-1,2-diylbis(sulfanediyl)dipropanoic acid (**2**).

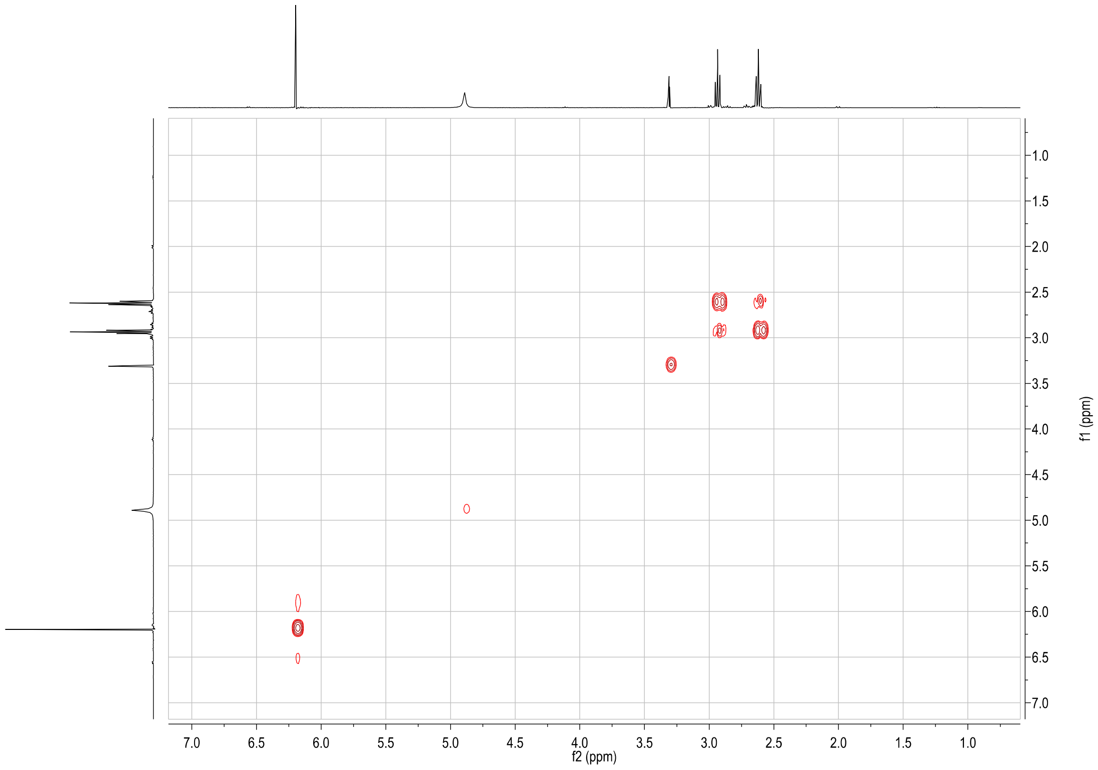


**Figure S3.** gHSQC spectra of (*Z*)-3,3′-(ethene-1,2-diylbis(sulfanediyl)dipropanoic acid (**2**).

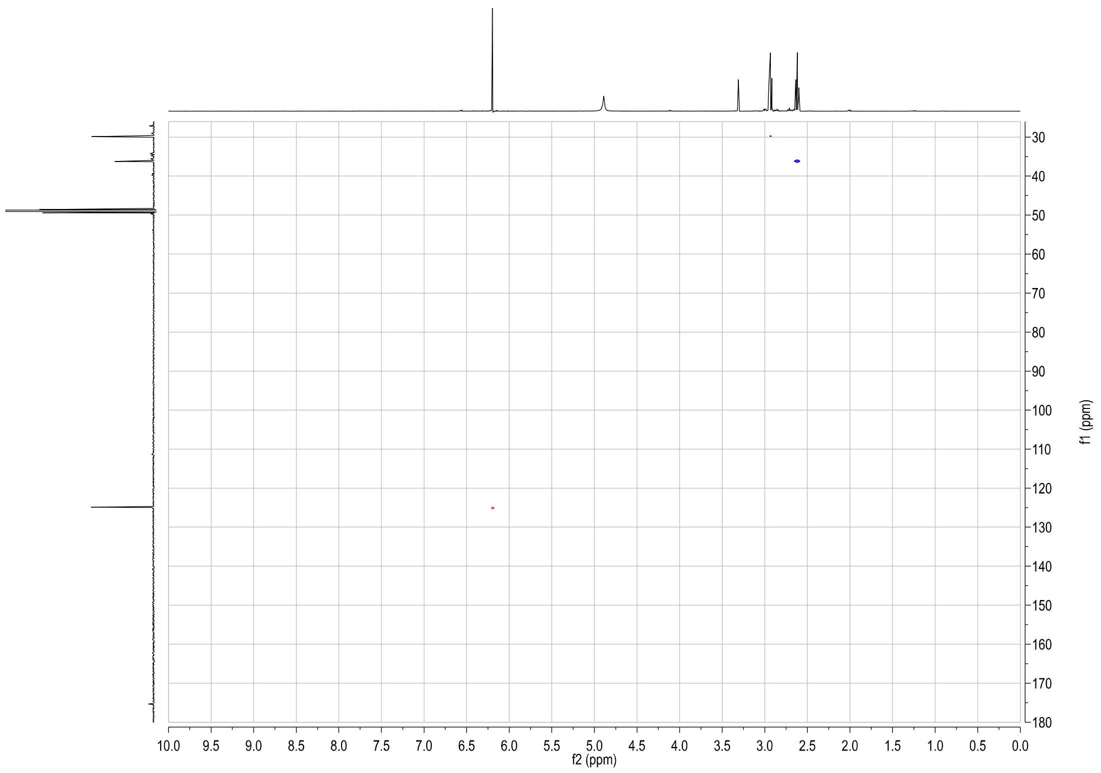


**Figure S4.** COSY spectra of (*Z*)-3,3′-(ethene-1,2-diylbis(sulfanediyl)dipropanoic acid (**2**).

## S2. Synthesis of non-targeted nanoparticles

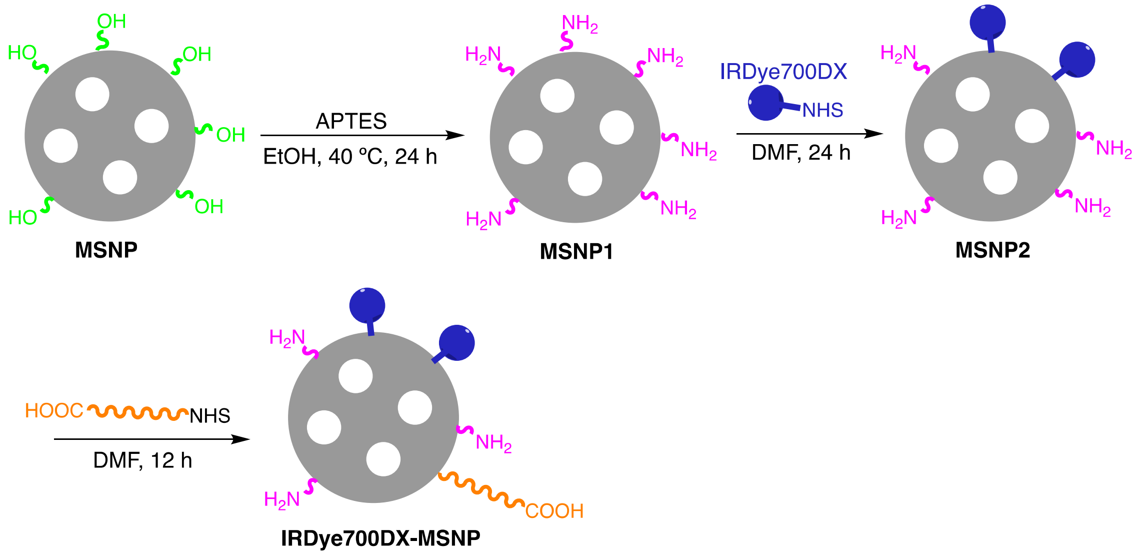


**Figure S5.** Preparation of the nanoparticle IRDye700DX-MSNP.

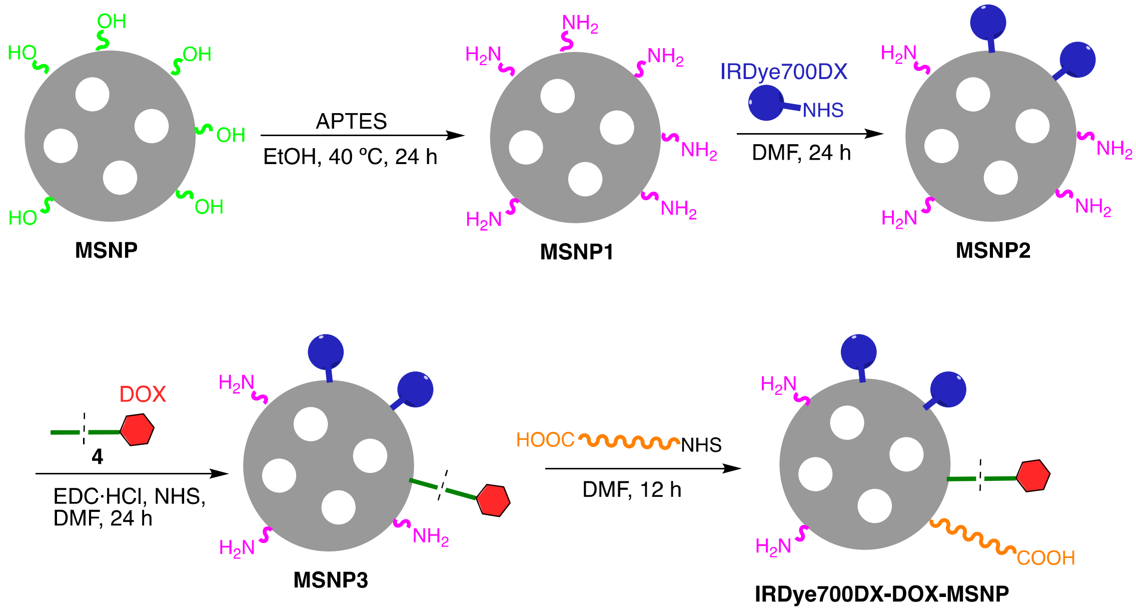


**Figure S6.** Preparation of the nanoparticle IRDye700-DOX-MSNP.

## S3. Synthesis of MSNP4 nanoparticles

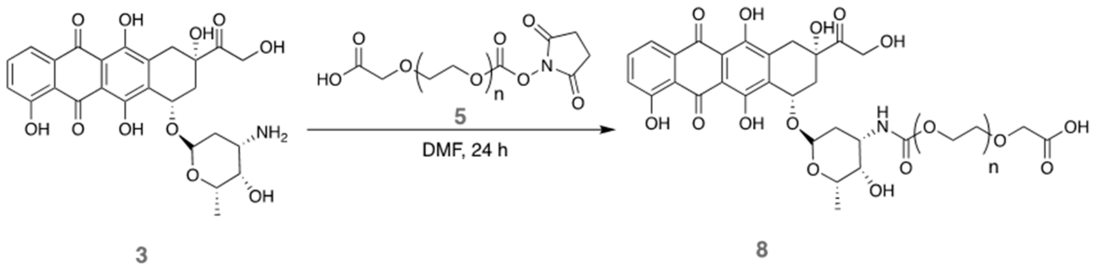


**Figure S7.** Preparation of the DOX-PEG conjugate. DOX was conjugated to an NHS-PEG$_{5kDa}$-COOH molecule via *N*-acylation.

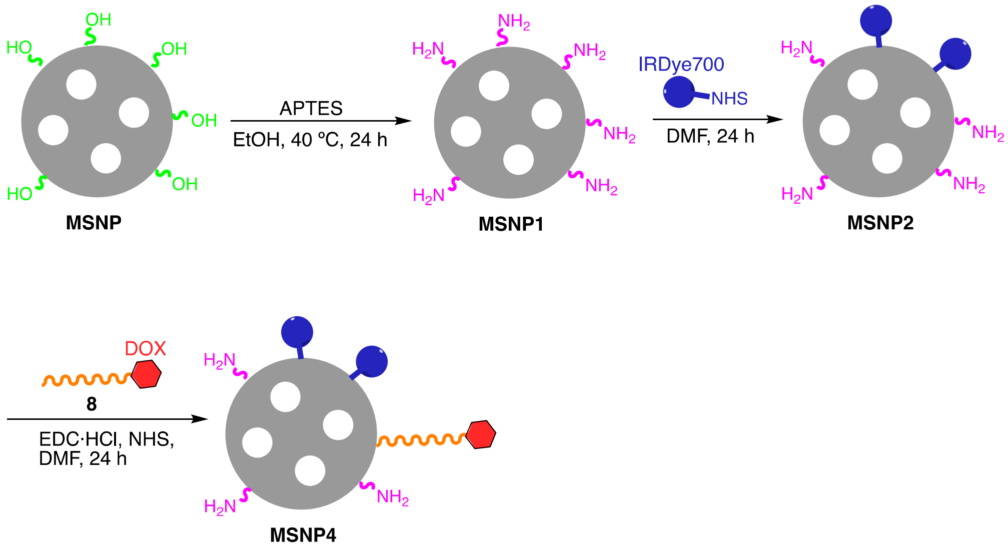


**Figure S8.** Preparation of the MSNP4 nanoparticles with covalently attached IRDye700DX and DOX-PEG$_{5kDa}$. The synthesis was performed in three steps: (i) Modification of the surface of MSNPs with amino groups (MSNP1). (ii) Attachment of IRDye700DX (MSNP2). (iii) Anchoring via *N*-acylation with EDC/NHS chemistry of the compound **8** to an amino group on surface of MSNP (MSNP4). MSNP4 was obtained with a DOX concentration of 110.3 μM.

## S4. Uptake of FITC-Cet-MSNPs in AsPC-1 and MIA PaCa-2 Cells

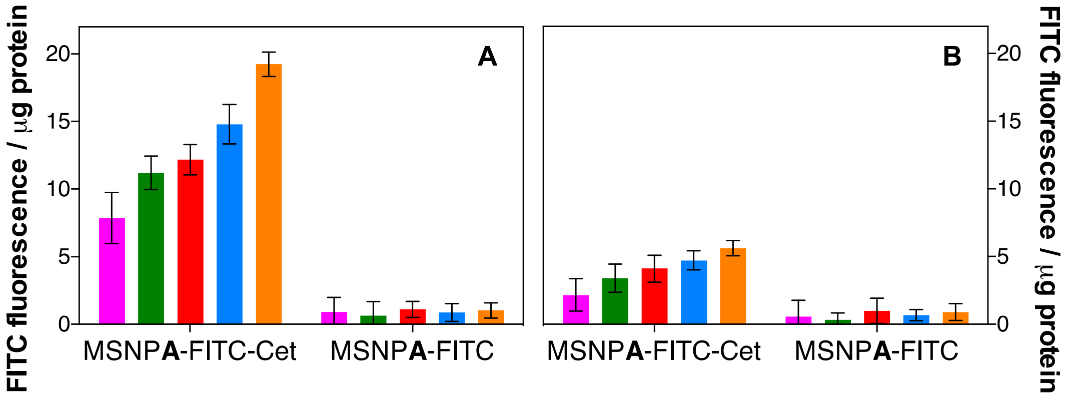


**Figure S9.** Cellular uptake of FITC-MSNP and FITC-cetuximab-MSNP nanoparticles in AsPC-1 (**A**) and MIA PaCa-2 (**B**) cells at different incubation times: 1 h (pink), 2 h (green), 4 h (red), 8 h (blue) and 24 h (orange). Cells were treated with the same concentration of FITC and MSNPs. Values reported are the mean ± SD of at least three independent experiments.

## S5. Cytotoxicity of Free IRDye700DX and Free DOX

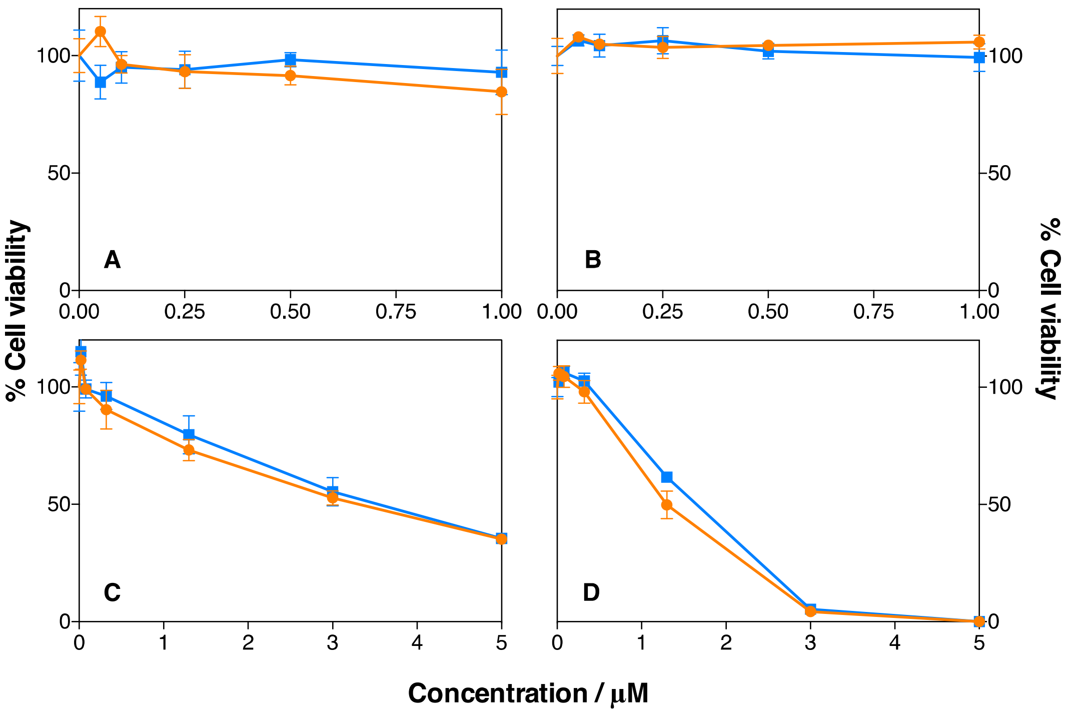


**Figure S10.** Phototoxicity studies of IRDye700DX (**A**,**B**) and doxorubicin (**C**,**D**) in AsPC-1 (**A**,**C**) and MIA PaCa-2 cells (**B**,**D**). Different concentrations of the drugs (0.05, 0.1, 0.25, 0.5, and 1 µM for IRDye700DX, and 0.02, 0.08, 0.32, 1.3, 3, and 5 µM for doxorubicin) were incubated for 24 h. Light treated cells are sown in orange (30 J·cm$^{-2}$, $\lambda_{exc}$ = 661 ± 10 nm) and dark controls in blue. The MTT assays were performed 24 h after the light treatments. Values reported are the mean ± SD of at least three independent experiments.

## S6. Cytotoxicity of free cetuximab

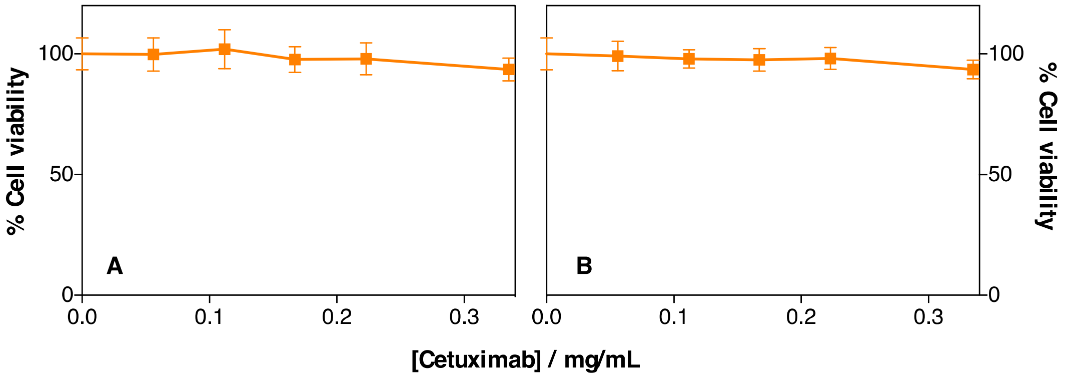


**Figure S11.** Phototoxicity studies of free Cetuximab in (**A**) AsPC-1 and (**B**) MIA PaCa-2 cells. A range of cetuximab concentrations (0.06, 0.11, 0.17, 0.22 and 0.33 mg/mL) were incubated in the cells for 24 h. Values reported are the mean ± SD of at least three independent experiments.

## S7. Thermodynamic Feasibility of Electron-Transfer Quenching of IRDye700DX by Doxorubicin

To assess the feasibility of electron-transfer between photoexcited IRDye700DX and doxorubicin, the free energy change was calculated using Equation (S1) [1]:

$$\Delta G^0 = F\left[E^0_{D^+/D} - E^0_{A/A^-}\right] - E^* - C \quad \text{(S1)}$$

where $F$ is Faraday's constant (96.5 kJ/mol), $E^0_{D^+/D}$ is the oxidation potential of the electron donor (IRDye700DX), $E^0_{A/A^-}$ the reduction potential of the acceptor (DOX), $E^*$ the excited-state energy of IRDye700DX and $C$ the coulombic stabilization energy of the radical ion pair, which can be neglected in aqueous solvents. Redox potentials are expressed in volt and energies in kJ/mol.

The oxidation potential of IRDye700DX can be assumed to be similar to that of other silicon phthalocyanines, $E^0_{D^+/D}$~0.95 V vs. Ag/AgCl in acetonitrile [2]. The reduction potential of DOX is $E^0_{A/A^-}$ = -0.665 vs. Ag/AgCl in aqueous solutions [3]. Neglecting to a first approximation any solvent effects on the redox potentials and taking $E^*$ = 173 kJ/mol for IRDye700DX in the singlet excited state, we estimate $\Delta G^0$~ -21 kJ/mol, which indicates that electron-transfer quenching of IRDye700DX singlet excited state is feasible, as observed. Quenching of the triplet excited state, on the other hand, should be less exergonic on account of the lower energy of this excited state, which is consistent with observing quenching in water but not in the less-polar solvents ethanol or dimethylformamide.

## References

1. Rehm, D.; Weller, A. Kinetics of Fluorescence Quenching by Electron and H-Atom Transfer. *Isr. J. Chem.* **1970**, *8*, 259–271, doi:10.1002/ijch.197000029.
2. Bandera, Y.; Burdette, M.; Shetzline, J.A.; Jenkins, R.; Creager, S.E.; Foulger, S. Synthesis of water soluble axially disubstituted silicon (IV) phthalocyanines with alkyne & azide functionality. *Dye Pigment.* **2016**, *125*, 72–79, doi:10.1016/j.dyepig.2015.10.007.
3. Guin, P.S.; Das, S. Exploration of Electrochemical Intermediates of the Anticancer Drug Doxorubicin Hydrochloride Using Cyclic Voltammetry and Simulation Studies with an Evaluation for Its Interaction with DNA. *Int. J. Electrochem.* **2014**, *2014*, 1–8, doi:10.1155/2014/517371.
